# Wogonoside alleviates hyperosmotic stress-induced inflammation and apoptosis in human corneal epithelial cells via PI3K/AKT signaling

**DOI:** 10.3389/fmed.2026.1828021

**Published:** 2026-05-04

**Authors:** Yuan Zhong, Jian Shi, Xi Long, Xiyuan Liu, Lihao Chen, Jun Peng, Qinghua Peng

**Affiliations:** 1 Hunan University of Chinese Medicine, Changsha, China; 2 Wenzhou Medical University Eye Hospital, Wenzhou, China

**Keywords:** dry eye, HCE-T cells, hyperosmotic stress, PI3K/AKT signaling pathway, wogonoside

## Abstract

**Background:**

Dry eye is a multifactorial ocular surface disorder in which tear hyperosmolarity acts as a major stressor that promotes inflammatory injury and apoptosis in corneal epithelial cells. Wogonoside (WGS), a flavonoid glycoside derived from *Scutellaria baicalensis*, has reported anti-inflammatory and cytoprotective properties; however, its role and mechanism in dry eye–related epithelial injury remain insufficiently defined.

**Methods:**

Network pharmacology was used to identify putative targets shared by WGS and dry eye, followed by pathway enrichment analysis. Molecular docking, molecular dynamics simulation, and cellular thermal shift assay (CETSA) were further used to evaluate the interaction between WGS and AKT1. Experimental validation was performed in transformed human corneal epithelial cells (HCE-T cells) exposed to hyperosmotic medium (500 mOsm). Cell viability, proliferation, apoptosis, inflammatory mediator levels, and PI3K/AKT signaling-related changes were assessed. To interrogate pathway involvement, cells were additionally treated with the AKT activator SC79 (10 μM) or the AKT inhibitor MK-2206 (2.5 μM).

**Results:**

A total of 203 overlapping targets were identified between WGS-related and dry eye-related target sets. Kyoto Encyclopedia of Genes and Genomes (KEGG) enrichment highlighted PI3K/AKT signaling as a potentially relevant pathway, and AKT1 was prioritized as a candidate target. Molecular docking and molecular dynamics simulation supported a stable interaction between WGS and AKT1, while CETSA provided additional evidence of AKT1 target engagement in cells. In hyperosmotic HCE-T cells, WGS improved cell viability and proliferative capacity, attenuated apoptosis, and reduced interleukin-1 beta (IL-1β), interleukin-6 (IL-6), and matrix metalloproteinase-9 (MMP-9) levels. These effects were accompanied by restoration of PI3K/AKT signaling. Mechanistically, MK-2206 partially attenuated the protective effects of WGS, whereas SC79 produced a similar protective profile, supporting a PI3K/AKT-dependent component.

**Conclusion:**

Wogonoside alleviates hyperosmotic stress-induced inflammatory injury and apoptosis in HCE-T cells. Integrated evidence from network pharmacology, molecular docking, molecular dynamics simulation, CETSA, and *in vitro* validation supports AKT1 as a candidate target of WGS. The protective effects of WGS were closely associated with restoration of PI3K/AKT signaling, suggesting that WGS may be a promising candidate for hyperosmotic stress-related corneal epithelial injury and merits further validation in appropriate animal models of dry eye.

## 1 Introduction

Dry eye is a prevalent ocular surface disorder characterized by disruption of tear film homeostasis, leading to ocular discomfort and impaired visual function (1). This condition substantially reduces quality of life and imposes a considerable socioeconomic burden (2). The TFOS DEWS III diagnostic methodology report identifies tear film instability and hyperosmolarity, ocular surface inflammation and damage, and neurosensory abnormalities as major drivers of disease onset and progression, and emphasizes that these factors can interact and perpetuate the disease process (3). Experimental and clinical evidence indicates that hyperosmotic stress disrupts corneal epithelial homeostasis, increases inflammatory mediator production, and promotes apoptosis, thereby compromising epithelial barrier integrity and delaying tissue repair (4, 5). Among the pathways regulating epithelial cell fate, the phosphoinositide 3-kinase (PI3K)/protein kinase B (AKT) signaling cascade plays a critical role in corneal epithelial cell survival, migration, and anti-apoptotic capacity (6). Consistent with this role, studies have shown that dry eye-related hyperosmolarity and oxidative stress suppress PI3K/AKT signaling, thereby amplifying inflammatory responses and apoptosis-related changes (7, 8). PI3K/AKT dysregulation is therefore increasingly regarded as an important molecular basis for ocular surface injury in dry eye (9, 10).

Artificial tears remain the mainstay of clinical management for dry eye, alleviating symptoms by supplementing and stabilizing the tear film (11). However, repeated use of preservative-containing formulations may cause ocular surface irritation or aggravate pre-existing epithelial damage (12). In patients with moderate to severe disease, lubrication alone is often insufficient to effectively control persistent inflammation or prevent progressive tissue injury (13). These limitations underscore the need for therapeutic strategies that combine anti-inflammatory and cytoprotective effects with good ocular surface tolerance (14).

Wogonoside (WGS), a major flavonoid glycoside derived from *Scutellaria baicalensis*, has attracted attention because of its anti-inflammatory and cytoprotective properties (15). In models of colitis and colitis-associated colorectal cancer, WGS markedly reduced the levels of pro-inflammatory cytokines, including interleukin-1 beta (IL-1β), interleukin-6 (IL-6), and tumor necrosis factor-alpha (TNF-*α*), and alleviated inflammation-related injury by inhibiting activation of the PI3K/AKT/nuclear factor-kappa B (NF-κB) axis (16). In a rat model of traumatic spinal cord injury, WGS improved motor function, reduced spinal cord water content, and suppressed proteins associated with the Toll-like receptor 4 (TLR4)/myeloid differentiation primary response 88 (MyD88)/NF-κB pathway, suggesting that it may mitigate tissue injury by suppressing inflammatory cascades (17). Together, these findings suggest that WGS has the potential to modulate inflammatory responses and attenuate inflammation-related tissue injury, although its role in dry eye has not yet been clarified.

In recent years, network pharmacology has been widely applied to elucidate the multi-target mechanisms of traditional Chinese medicines and their active constituents (18). By integrating compound-target-pathway-disease networks, this approach helps identify key signaling axes for subsequent experimental validation (19). In the present study, PI3K/AKT signaling was identified as a candidate pathway potentially involved in the effects of WGS on hyperosmotic stress-related corneal epithelial injury. A hyperosmotic HCE-T cell model was then used to evaluate the protective effects of WGS on corneal epithelial cells and the involvement of this pathway.

## 2 Materials and methods

### 2.1 Network pharmacology analysis and *in silico* validation

#### 2.1.1 Gene collection of dry eye

Dry eye-related genes were retrieved from GeneCards, the Therapeutic Target Database (TTD), and Online Mendelian Inheritance in Man (OMIM) using “dry eye” as the search term (20–22). The GeneCards, TTD, and OMIM databases were accessed on 14 July 2025 at the following URLs: https://www.genecards.org, https://db.idrblab.net/ttd/, and https://omim.org, respectively. For GeneCards, targets with a relevance score of at least 1.00 were retained. Targets obtained from TTD and OMIM were included directly. The targets from these three databases were then merged, and duplicate entries were removed.

#### 2.1.2 Prediction of potential WGS targets

Potential targets of WGS were predicted using PharmMapper and SwissTargetPrediction (23, 24). PharmMapper and SwissTargetPrediction were accessed on 17 July 2025 at http://www.lilab-ecust.cn/pharmmapper/ and http://www.swisstargetprediction.ch, respectively. The structural information of WGS was prepared and submitted according to the standard workflows of the two platforms. For both platforms, the species was restricted to *Homo sapiens*. For SwissTargetPrediction, only targets with probability > 0 were retained, whereas for PharmMapper, only targets with a Fit Score > 2.0 were retained. The target lists obtained from PharmMapper and SwissTargetPrediction were then merged, and duplicate entries were removed.

#### 2.1.3 Screening of common targets and construction of the WGS-target-dry eye network

To ensure consistent nomenclature, targets from both the WGS target set and the dry eye-related gene set were standardized using the UniProt database (https://www.uniprot.org, accessed on 20 July 2025) (25). After standardization and removal of duplicate entries, the intersection between dry eye-related genes and the predicted targets of WGS was defined as the set of overlapping targets. A Venn diagram was generated using the Venny platform (https://bioinfogp.cnb.csic.es/tools/venny/, accessed on 24 July 2025) to visualize the shared targets. The overlapping targets were then imported into Cytoscape (version 3.8.0) to construct and visualize the WGS-target-dry eye network (49, 50). AKT1 was subsequently prioritized for molecular docking and experimental validation on the basis of KEGG pathway enrichment together with protein–protein interaction network analysis.

#### 2.1.4 Protein–protein interaction (PPI) network construction

The overlapping targets were submitted to the STRING database (https://www.string-db.org/, accessed on 24 July 2025) to construct a protein–protein interaction (PPI) network (26). The species was restricted to *Homo sapiens*. The resulting interaction data were downloaded and imported into Cytoscape (version 3.8.0) for network visualization and topological analysis.

#### 2.1.5 GO functional annotation and KEGG pathway enrichment analysis

Gene Ontology (GO) functional annotation and Kyoto Encyclopedia of Genes and Genomes (KEGG) pathway enrichment analyses were performed for the overlapping targets using the Metascape platform (https://metascape.org, accessed on 22 July 2025) (27). GO enrichment results were classified into biological process (BP), molecular function (MF), and cellular component (CC) categories, following the Gene Ontology framework (28). KEGG pathway interpretation was based on the KEGG pathway resource (29). Bar plots were generated to visualize the GO enrichment results, and bubble plots were used to present the KEGG pathway enrichment results. Chord diagrams were additionally generated to illustrate the relationships between representative enriched pathways and their corresponding targets. For KEGG pathway enrichment, multiple testing-corrected statistics provided in the original output, including Benjamini-adjusted *p*-values and FDR, were examined and used as reference for pathway interpretation and prioritization.

#### 2.1.6 Molecular docking

To evaluate the binding mode of Wogonoside (WGS) toward AKT1, molecular docking was performed using smina, a fork of AutoDock Vina derived from version 1.1.2 and developed for improved scoring and minimization (30, 31). The three-dimensional structure of AKT1 was obtained from the AlphaFold Protein Structure Database (model ID: AF-P31749-F1-v6) (32), and the three-dimensional structure of WGS was downloaded from PubChem in SDF format (33). Prior to docking, the receptor structure was subjected to standard preprocessing, including hydrogen addition and conversion to the docking input format. The ligand structure was energy-minimized and prepared for docking on the basis of the downloaded 3D SDF file. The docking grid box was centered at *x* = −2.3545, *y* = −9.5940, and *z* = 6.5915, with dimensions of 15.439 × 11.060 × 12.077 Å, covering the kinase-domain binding pocket of AKT1. Docking poses were ranked using the default smina scoring function, and the conformation with the lowest docking score was selected for subsequent visualization and interaction analysis. Because the receptor structure was obtained from the AlphaFold Protein Structure Database rather than a co-crystallized ligand-bound complex, conventional redocking validation was not performed.

#### 2.1.7 Molecular dynamics simulation

To further evaluate the dynamic stability of the AKT1-WGS complex after docking, molecular dynamics (MD) simulation was performed using GROMACS 2023.2 (34). The AKT1 protein was described using the amber99sb-ildn force field (35), whereas WGS was parameterized using the general AMBER force field (GAFF) (36). The protein-ligand complex was placed in a cubic periodic box with a minimum distance of 1.0 nm between the complex and the box edge. The system was solvated using the TIP3P water model, and Na^+^ and Cl^−^ ions were added to neutralize the system and achieve an ionic concentration of 0.15 M. Energy minimization was performed using the steepest descent algorithm until the maximum force was reduced to below 500 kJ/mol/nm. After minimization, the system was sequentially equilibrated under the NVT and NPT ensembles, followed by a 100 ns production run comprising 5,000,000 steps with an integration time step of 2 fs. The short-range cutoff distances for both van der Waals and electrostatic interactions were set to 1.2 nm. Van der Waals interactions were treated using the force-switch scheme with a switching distance of 1.0 nm, whereas long-range electrostatic interactions were calculated using the particle mesh Ewald method. Temperature was controlled using the V-rescale thermostat at 300 K with a coupling constant of 0.1 ps. For pressure coupling, the Berendsen barostat was used during the NPT equilibration stage, followed by the Parrinello-Rahman barostat during the production run, with a reference pressure of 1.0 bar and a coupling constant of 2.0 ps. All simulations were independently repeated three times with different initial random seeds.

Trajectory analyses were performed using the built-in GROMACS utilities. The root mean square deviation (RMSD), root mean square fluctuation (RMSF), radius of gyration (Rg), solvent-accessible surface area (SASA), and intermolecular hydrogen bonds were calculated to assess the conformational stability and interaction persistence of the AKT1-WGS complex. In addition, the free energy landscape (FEL) was constructed based on the RMSD and Rg reaction coordinates, and binding free energy was further estimated using the MM/PBSA method (37).

### 2.2 *In vitro* experimental validation

#### 2.2.1 Cell culture and establishment of the hyperosmotic stress model

Transformed human corneal epithelial cells (HCE-T; batch no. 20240514) were purchased from Guangzhou Cellcook Biotech Co., Ltd. (Guangzhou, China). Cells were cultured in DMEM/F12 complete medium supplemented with 10% fetal bovine serum, penicillin–streptomycin, epidermal growth factor (EGF), and insulin at 37 °C in a humidified incubator containing 5% CO₂. Cells were routinely passaged with trypsin at 80–90% confluence. An *in vitro* dry eye-related hyperosmotic stress model was established by increasing the osmolarity of the culture medium with NaCl supplementation (Sinopharm, Shanghai, China; cat. no. 10019318). Hyperosmotic conditions were optimized in preliminary CCK-8 assays, and 500 mOsm was selected for subsequent experiments because it produced a stable and reproducible cellular injury phenotype suitable for mechanistic investigations (7).

**Figure 1:**
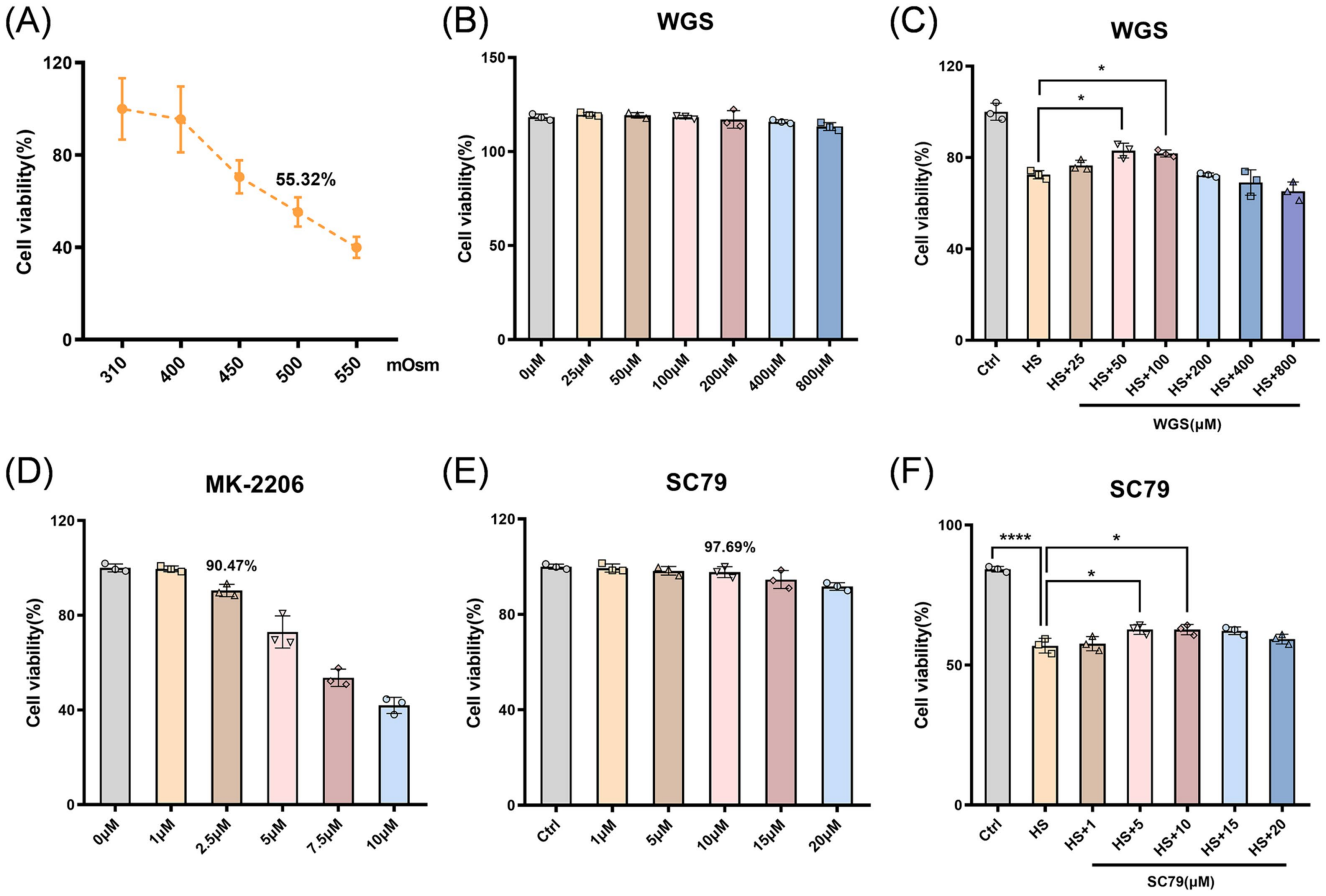
CCK-8 assay for determining the hyperosmotic condition and working concentrations of WGS and AKT modulators in HCE-T cells. **(A)** Cell viability under increasing medium osmolarity. Cell viability progressively decreased as osmolarity increased and reached 55.32% at 500 mOsm; therefore, 500 mOsm was selected for subsequent experiments. **(B)** Cell viability after treatment with WGS alone under isotonic conditions, showing no obvious cytotoxicity within the tested concentration range. **(C)** Cell viability under 500 mOsm hyperosmotic stress after treatment with different concentrations of WGS. **(D)** Cell viability after treatment with different concentrations of MK-2206. **(E)** Cell viability after treatment with different concentrations of SC79 under isotonic conditions. **(F)** Cell viability under hyperosmotic stress after treatment with different concentrations of SC79. Three replicate wells were set for each group, and the mean value was used for analysis. Data are presented as mean ± standard deviation (SD) and analyzed by one-way ANOVA followed by Tukey’s multiple-comparisons test. ns, not significant; *p < 0.05, **p < 0.01, ***p < 0.001, ****p < 0.0001.

To determine the appropriate working concentrations of WGS, a stepwise screening strategy was used. Under isotonic conditions, cells were first treated with WGS at concentrations of 25–800 μM for 24 h, and cell viability was assessed by CCK-8 assay to determine the *in vitro* tolerance range. Subsequently, dose selection was performed under hyperosmotic stress: cells were exposed to 500 mOsm hyperosmotic medium for 24 h, followed by replacement with isotonic complete medium containing different concentrations of WGS (25–800 μM) for an additional 24 h to evaluate its protective effects after stress induction, as described in a similar post-stress intervention design in corneal epithelial cells (9). Based on the screening results, 25, 50, and 100 μM were selected as the low-, medium-, and high-dose concentrations for subsequent dose-dependent experiments, and 50 μM was used in the downstream mechanistic studies. The working concentrations of the AKT inhibitor MK-2206 (2.5 μM) and the AKT activator SC79 (10 μM) were determined by preliminary CCK-8 assays (see Results Section 3.3.1 and Figures 1D–F) (5, 38).

#### 2.2.2 Experimental design and treatment protocols

The in vitro experiments were conducted in two sequential phases. The first phase was designed to establish the hyperosmotic stress condition and determine the working concentrations of WGS based on CCK-8 results. The second phase was designed to evaluate the involvement of PI3K/AKT signaling by pharmacological modulation using the AKT activator SC79 (MCE, Monmouth Junction, USA; cat. no. HY-18794) and the AKT inhibitor MK-2206 (MCE, Monmouth Junction, USA; cat. no. HY-10358), together with WGS (Yuanye, Shanghai, China; cat. no. B20488), under hyperosmotic stress.

In the first phase, cells were divided into five groups: Control, HS, HS + WGS (25 μM), HS + WGS (50 μM), and HS + WGS (100 μM). In the second phase, cells were divided into six groups: Control, HS, HS + WGS (50 μM), HS + SC79, HS + MK-2206, and HS + MK-2206 + WGS. Control cells were maintained in isotonic medium for 48 h. In the HS group, cells were exposed to 500 mOsm hyperosmotic medium for 24 h, followed by replacement with fresh isotonic complete medium for another 24 h. In the intervention groups, WGS, SC79, MK-2206, or MK-2206 combined with WGS was added during the second 24 h period after replacement with isotonic medium. This design was intended to evaluate the effects of these interventions after hyperosmotic injury had been established.

#### 2.2.3 CCK-8 assay

Cell viability was assessed using the Cell Counting Kit-8 (CCK-8) assay. HCE-T cells were seeded into 96-well plates at a density of 5 × 10^3^ cells per well in 100 μL of medium, with three replicate wells for each group. After the indicated treatments, the medium was replaced with complete medium containing CCK-8 reagent (Abiowell, Changsha, China; cat. no. AWC0114a; 10 μL per well), and the cells were incubated for 4 h at 37 °C in a direct-heat CO₂ incubator (SANTN, Shanghai, China; model no. DH-160I). Absorbance at 450 nm was measured using a multifunctional microplate reader (Huisong, Shenzhen, China; model no. MB-530), and the mean OD450 value of the replicate wells was used for analysis. Cell viability was normalized to the Control group and expressed as a percentage. The CCK-8 assay was also used in preliminary experiments to determine the hyperosmotic stress condition (500 mOsm) and to optimize the working concentrations of WGS, SC79, and MK-2206.

#### 2.2.4 5-ethynyl-2′-deoxyuridine (EdU) incorporation assay

Cell proliferation was evaluated using an EdU incorporation assay with an EdU detection kit (RiboBio, Guangzhou, China; cat. no. C10310). After the indicated treatments, cells were incubated with EdU working solution, fixed, and permeabilized according to the manufacturer’s instructions. Incorporated EdU was detected using the click-reaction staining solution provided in the kit, and nuclei were counterstained with Hoechst 33342. Images were acquired using an inverted biological microscope (Zhongxian Hengye, Beijing, China; model no. DSZ2000X). Cell proliferation was quantified as the percentage of EdU-positive cells among total Hoechst-stained cells.

#### 2.2.5 Flow cytometric analysis of apoptosis

Apoptosis was analyzed by flow cytometry using Annexin V-APC/propidium iodide (PI) staining with an apoptosis detection kit (KeyGEN BioTECH, Nanjing, China; cat. no. KGA1107). After the indicated treatments, cells were harvested using EDTA-free trypsin solution (Abiowell, Changsha, China; cat. no. AWC0236), washed twice with PBS, and collected by centrifugation at 1500 rpm for 5 min each time. Cells were resuspended in 500 μL of binding buffer, stained with 5 μL Annexin V-APC and 5 μL PI, and incubated for 10 min at room temperature in the dark. Samples were analyzed within 1 h using a flow cytometer (Beckman Coulter, Brea, United States; model no. A00-1-1102).

#### 2.2.6 TUNEL assay

Cells cultured on coverslips were washed three times with PBS and fixed with 4% paraformaldehyde. After permeabilization with immunostaining permeabilization solution [0.3% Triton X-100; Abiowell, Changsha, China; cat. no. AWI0603a] for 20 min at room temperature, cells were washed three times with PBS for 5 min each. The TUNEL reaction mixture from a TUNEL kit (Yeasen, Shanghai, China; cat. no. 40306ES50) was then added to completely cover the cells, followed by incubation at 37 °C in the dark for 2 h. After washing with PBS, nuclei were counterstained with DAPI (Abiowell, Changsha, China; cat. no. AWI0331a) for 5 min in the dark. Coverslips were mounted with buffered glycerol (Abiowell, Changsha, China; cat. no. AWI0168a) and imaged using a fluorescence microscope (Motic, Xiamen, China; model no. BA210T). Apoptosis was quantified as the percentage of TUNEL-positive cells.

#### 2.2.7 Immunofluorescence staining

Immunofluorescence staining was performed to detect phosphorylated PI3K (p-PI3K) and phosphorylated AKT [p-AKT (Ser473)]. Cells cultured on coverslips were fixed with 4% paraformaldehyde, permeabilized with immunostaining permeabilization solution [0.3% Triton X-100; Abiowell, Changsha, China; cat. no. AWI0603a], and blocked with 5% BSA. Cells were then incubated overnight at 4 °C with primary antibodies against p-PI3K (Thermo Fisher Scientific, United States; cat. no. PA5-99367; 1:50) and p-AKT (Ser473) (Abiowell, Changsha, China; cat. no. AWA40438; 1:50), followed by incubation with Goat anti-Rabbit IgG-488 secondary antibody (Abiowell, Changsha, China; cat. no. AWS0005; 1:200). Nuclei were counterstained with DAPI (Abiowell, Changsha, China; cat. no. AWI0331a), and images were acquired using a fluorescence microscope (Motic, Xiamen, China; model no. BA210T).

#### 2.2.8 Enzyme-linked immunosorbent assay (ELISA)

Culture supernatants were collected after treatment and centrifuged using a benchtop refrigerated centrifuge (Xiangyi, Changsha, China; model no. H1650R) to remove cell debris. The levels of IL-6, matrix metalloproteinase-9 (MMP-9), and IL-1β were measured using commercial ELISA kits according to the manufacturers’ instructions: IL-6 ELISA kit (Proteintech, Rosemont, United States; cat. no. KE00139), MMP-9 ELISA kit (Proteintech, Rosemont, United States; cat. no. KE00456; lot no. 40002573), and IL-1β ELISA kit (Proteintech, Rosemont, United States; cat. no. KE00021). After plate washing with an automated microplate washer (Huisong, Shenzhen, China; model no. PW-812), absorbance was measured using a multifunctional microplate reader (Huisong, Shenzhen, China; model no. MB-530), and concentrations were calculated from standard curves.

#### 2.2.9 Reverse transcription quantitative polymerase chain reaction (RT-qPCR)

Total RNA was extracted from cells using TRIzol reagent (Thermo Fisher Scientific, Waltham, United States; cat. no. 15596026), and RNA purity was assessed by UV spectrophotometry based on the A260/A280 ratio. Equal amounts of RNA were reverse-transcribed into cDNA using a reverse transcription kit (Kangwei Century, Beijing, China; cat. no. CW2569) in a 20 μL reaction volume at 50 °C for 50 min, followed by 85 °C for 5 min. Quantitative PCR was performed using a SYBR Green-based mix (UltraSYBR Mixture; Kangwei Century, Beijing, China; cat. no. CW2601) on a QuantStudio 1 Real-Time PCR System (Applied Biosystems, Waltham, United States), with β-actin as the internal reference. For RT-qPCR, three independent biological samples were included in each group, and each sample for each target gene was analyzed in triplicate using optical consumables (Thermo Fisher Scientific, Waltham, United States; cat. no. SPL0960). The mean Ct value of the technical replicates was used for subsequent analysis. The amplification program consisted of 95 °C for 10 min, followed by 40 cycles of 95 °C for 15 s and 60 °C for 30 s, and a melting-curve step from 60 °C to 95 °C. Relative mRNA expression levels of PI3K, AKT, Bax, and Bcl-2 were calculated using the comparative Ct (2^−ΔΔCt) method and normalized to β-actin (39). Primer sequences are listed in Table 1.

**Table 1:** Primer sequence information.

Primer name	Sequence (5′–3′)	Amplicon length (bp)
PI3K	F TGCGTCTACTAAAATGCATGG	122 bp
	R AACTGAAGGTTAATGGGTCA	
AKT	F AGCCCTGGACTACCTGCACTCG	98 bp
	R CTGTGATCTTAATGTGCCCGTCCT	
Bax	F TCACTGAAGCGACTGATGTCCC	96 bp
	R ACTCCCGCCACAAAGATGGTC	
Bcl-2	F AGCTGCACCTGACGCCCTT	147 bp
	R ACATCTCCCGGTTGACGCTCT	
β-actin	F ACCCTGAAGTACCCCATCGAG	224 bp
	R AGCACAGCCTGGATAGCAAC	

#### 2.2.10 Western blot

Western blot analysis was performed as described previously with minor modifications (40). Cells were collected, and total protein was extracted using RIPA lysis buffer (Abiowell, Changsha, China; cat. no. AWB0136). Lysates were centrifuged at 12,000 rpm for 15 min at 4 °C, and the supernatants were collected. Protein concentrations were determined using a BCA protein assay kit (Abiowell, Changsha, China; cat. no. AWB0104). Equal amounts of protein were denatured, separated by SDS-PAGE, and transferred onto PVDF membranes (Millipore, Burlington, United States) at a constant current of 300 mA using a wet transfer system (Liuyi, Beijing, China; model no. DYCZ-40D). Membranes were blocked with non-fat milk and incubated overnight at 4 °C with primary antibodies against PI3K (1:5000; Proteintech, Rosemont, United States; cat. no. 60225-1-Ig), p-PI3K (1:1000; Cell Signaling Technology, Danvers, United States; cat. no. 17366), AKT (1:500; Abiowell, Changsha, China; cat. no. AWA10126), p-AKT (Ser473) (1:1000; Abiowell, Changsha, China; cat. no. AWA10017), cleaved caspase-3 (1:500; Proteintech, Rosemont, United States; cat. no. 19677-1-AP), Bax (1:1000; Abiowell, Changsha, China; cat. no. AWA58069), Bcl-2 (1:1000; Abiowell, Changsha, China; cat. no. AWA00392), p-NF-κB p65 (1:500; Abiowell, Changsha, China; cat. no. AWA47474), IκBα (1:1000; Cell Signaling Technology, Danvers, United States; cat. no. 4814S), and β-actin (1:5000; Proteintech, Rosemont, United States; cat. no. 66009-1-Ig). Membranes were then incubated with the corresponding HRP-conjugated secondary antibodies (Abiowell, Changsha, China; cat. Nos. AWS0001 and AWS0002) for 90 min at room temperature and washed three times with PBST for 15 min each. Signals were developed using an ECL substrate and detected with a chemiluminescence imaging system (Qinxiang, Shanghai, China; model no. ChemiScope 6,100). Band intensities were quantified using ImageJ. Phosphorylation levels were expressed as the p-PI3K/PI3K and p-AKT/AKT ratios.

#### 2.2.11 Cellular thermal shift assay (CETSA)

To further assess whether WGS engages AKT1 in cells, a cellular thermal shift assay (CETSA) was performed in HCE-T cells, as previously described for cellular target engagement analysis (41). Cells were first exposed to 500 mOsm hyperosmotic medium for 24 h and then treated with WGS (50 μM) or an equal volume of DMSO for an additional 24 h. At the end of treatment, cells were collected and equally distributed into PCR tubes for heat treatment at 37, 42, 46, 50, 54, 58, and 62 °C.

After heating for 3 min at the indicated temperatures, samples were immediately cooled on ice for 3–5 min. Cells were then lysed on ice in a non-denaturing NP-40-containing lysis buffer for 20–30 min with intermittent gentle mixing. Lysates were centrifuged at 20,000 × g for 15–20 min at 4 °C, and the soluble fractions were collected for Western blot analysis. Soluble AKT1 levels were detected, with GAPDH used as the internal reference.

For quantitative analysis, the band intensity of soluble AKT1 at each temperature [I(T)] was normalized to that at 37 °C [I(37 °C)] within the same treatment group, and the normalized soluble fraction was calculated as I(T)/I(37 °C). Melting curves were generated by plotting the normalized soluble AKT1 fraction against temperature. The apparent melting temperature (Tm) was obtained by nonlinear curve fitting, and the thermal stabilization effect of WGS on AKT1 was evaluated by comparing the Tm values between the DMSO and WGS groups.

#### 2.2.12 Statistical analysis

Data are presented as mean ± standard deviation (SD). Statistical analyses were performed using GraphPad Prism (version 8.0). Comparisons among multiple groups were performed using one-way analysis of variance (ANOVA), followed by Tukey’s multiple-comparisons test. A two-sided *p*-value < 0.05 was considered statistically significant.

## 3 Results

### 3.1 Network pharmacology analysis

#### 3.1.1 Identification of potential targets

**Figure 2:**
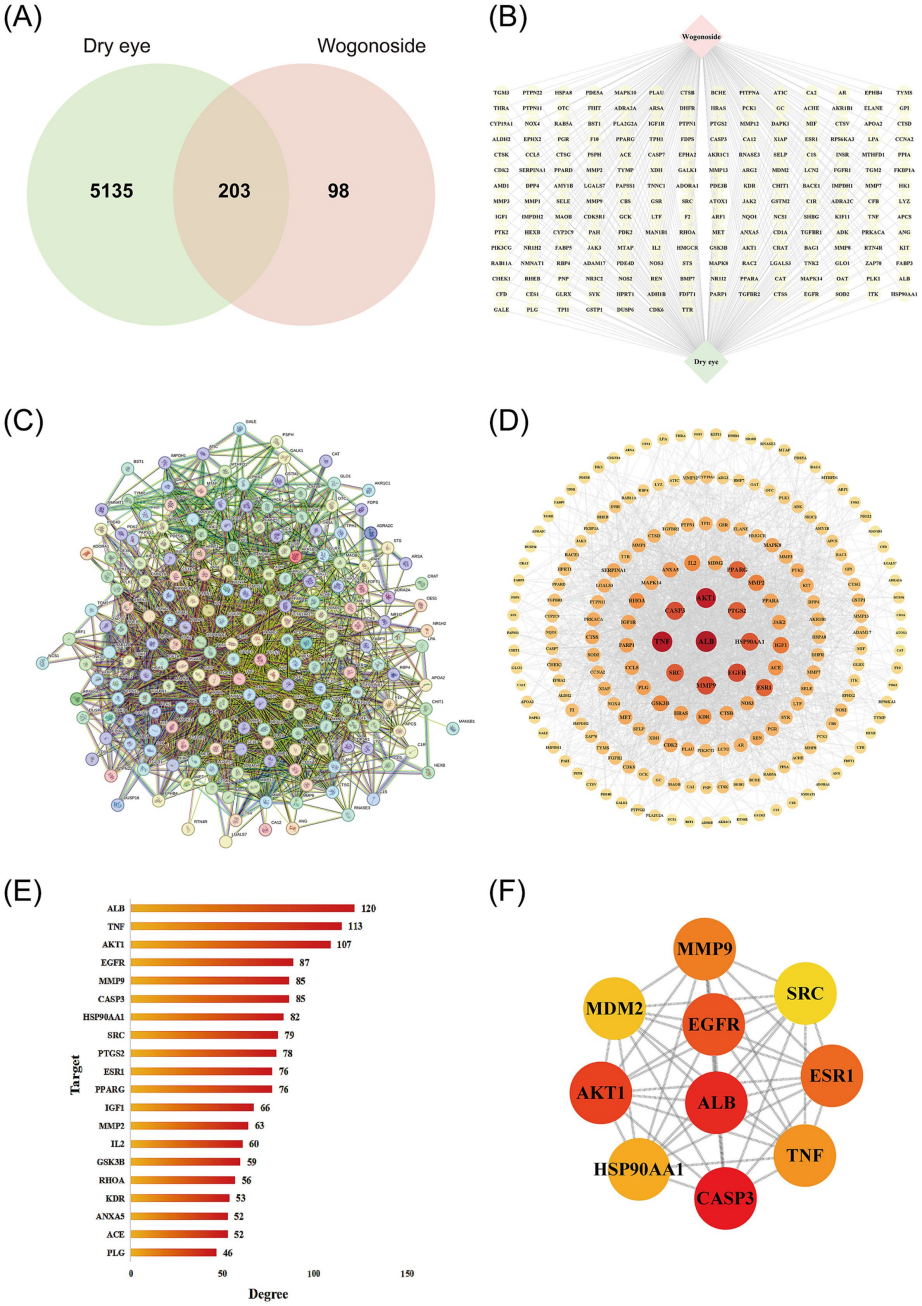
Network pharmacology analysis of WGS in dry eye. **(A)** Venn diagram showing the overlap between dry eye-related targets and WGS-related targets. **(B)** WGS-target-dry eye network illustrating the multi-target characteristics of WGS in dry eye. In this network, the red node represents WGS, the green node represents dry eye, and the yellow nodes represent the overlapping targets. Edges indicate predicted interactions among nodes. **(C)** Protein–protein interaction (PPI) network of the overlapping targets constructed using the STRING database. **(D)** Cytoscape visualization of the PPI network. Node size and color intensity reflect topological importance within the network. **(E)** Top 20 degree-ranked targets in the PPI network. The horizontal axis represents degree values, and the vertical axis represents target names. **(F)** Top 10 hub genes identified by the cytoHubba MCC algorithm. Darker node color indicates higher topological importance.

A total of 303 potential targets of WGS were identified. Specifically, 288 targets were obtained from PharmMapper and 19 from SwissTargetPrediction. After merging the two target lists and removing duplicate entries, 303 non-redundant WGS-related targets were retained. A total of 5,338 dry eye-related targets were collected. Specifically, 5,199 targets were retrieved from GeneCards using a relevance score threshold of ≥1.00, together with additional targets from TTD (*n* = 6) and OMIM (*n* = 159). After integration of the three datasets and removal of duplicate entries, 5,338 non-redundant dry eye-related targets were retained. Intersection of the WGS-related targets with the dry eye-related targets yielded 203 overlapping targets, as shown in the Venn diagram (Figure 2A). The WGS-target-dry eye network is presented in Figure 2B.

#### 3.1.2 PPI network of overlapping targets between WGS and dry eye

The 203 overlapping targets between WGS and dry eye were imported into the STRING database (version 12.0). Because not all targets could be successfully mapped in STRING and Metascape using standardized identifiers, 199 targets were retained for downstream protein–protein interaction and enrichment analyses. The resulting protein–protein interaction network contained 199 nodes and 2,439 edges, with an average degree of 24.51 (Figure 2C). These targets were subsequently imported into Cytoscape (version 3.8.0) for visualization and topological analysis of the PPI network (Figure 2D). In this network, nodes represent individual targets and edges represent interactions, whereas nodes with higher degree values are displayed as larger and darker. The top 20 degree-ranked targets were identified, and the cytoHubba MCC algorithm was further applied to screen the top 10 hub genes (Figures 2E,F).

#### 3.1.3 GO and KEGG enrichment analyses

**Figure 3:**
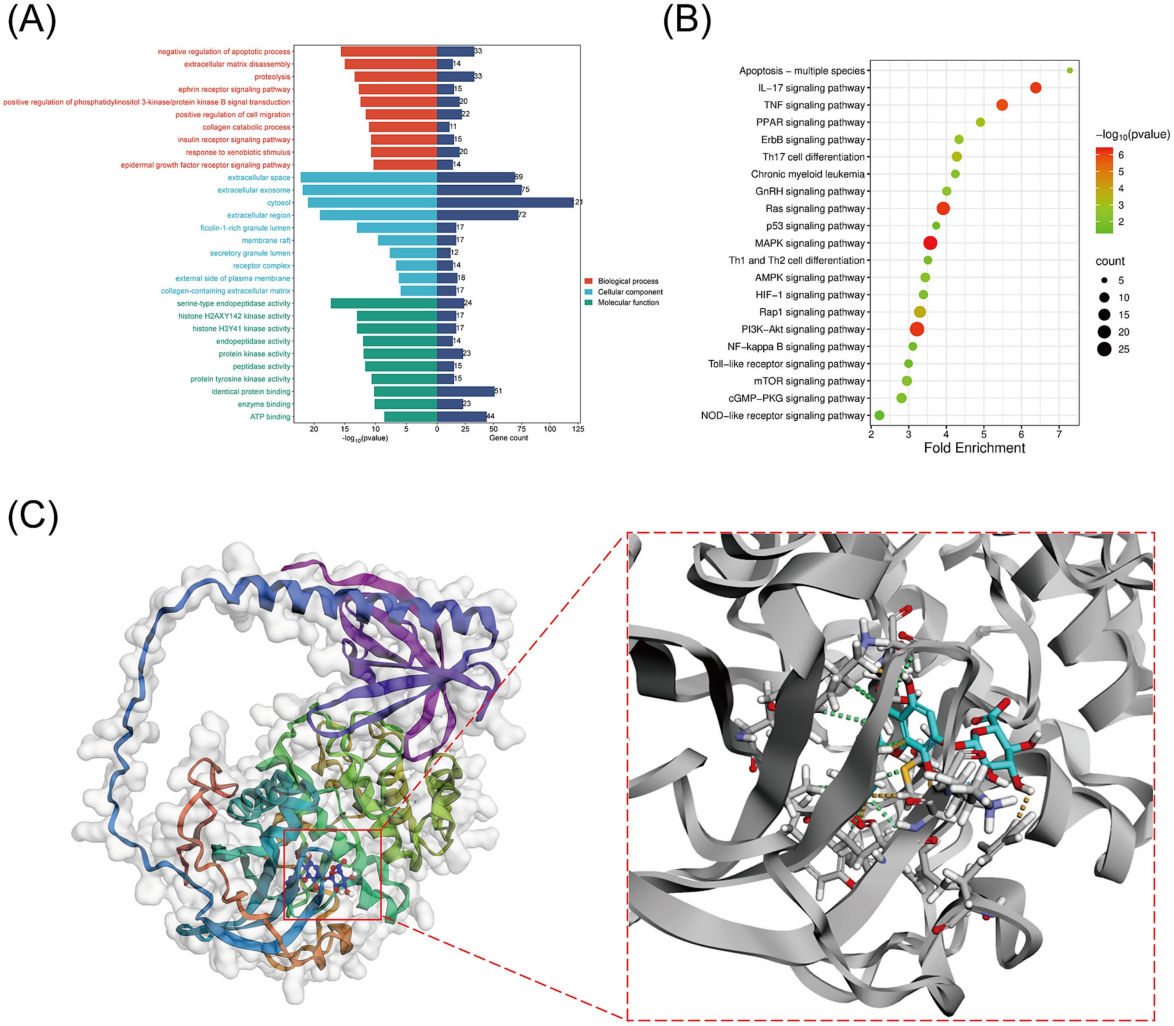
Functional enrichment analysis of overlapping targets and molecular docking validation. **(A)** GO enrichment analysis of the overlapping targets. Enriched GO terms are shown for biological process (BP, red), cellular component (CC, blue), and molecular function (MF, green). The left *x*-axis indicates −log10(*p*-value), and the right *x*-axis indicates gene count. Each row represents one GO term. **(B)** KEGG pathway enrichment analysis of the overlapping targets. The *x*-axis indicates fold enrichment, and the *y*-axis lists the enriched pathways. Dot size represents gene count, and dot color indicates −log10(*p*-value), as shown in the color scale. Multiple testing-corrected statistics, including Benjamini-adjusted *p*-values and FDR, were additionally used for pathway interpretation. **(C)** Representative three-dimensional docking pose of WGS within the AKT1 kinase-domain binding pocket, showing the predicted binding conformation and key interacting residues.

Of the 203 overlapping targets identified, 199 could be mapped with standardized identifiers and were therefore retained for GO functional annotation and KEGG pathway enrichment analyses in Metascape. GO annotation was performed across the biological process (BP), cellular component (CC), and molecular function (MF) categories. The original Metascape output contained 522 BP terms, 64 CC terms, and 170 MF terms. The top 10 terms in each category are shown in Figure 3A and are presented here as descriptive functional annotation results. Among these, multiple GO terms were associated with cellular stress responses, inflammatory regulation, and signal transduction.

The KEGG pathway enrichment analysis identified 154 enriched pathways in the original output. As shown in Figure 3B, the *x*-axis represents fold enrichment and the *y*-axis lists the enriched pathways, whereas dot size indicates gene count and dot color represents -log10(*p*-value). Multiple testing-corrected statistics were available in the original KEGG output, including Bonferroni, Benjamini, and FDR values. Among the enriched pathways, the PI3K/Akt signaling pathway remained significant after correction (raw *p* = 6.63 × 10^−7^, Benjamini-adjusted *p* = 1.33 × 10^−5^, FDR = 7.59 × 10^−6^) and was therefore highlighted as a potentially relevant pathway associated with the protective effects of WGS in dry eye. Combined with network topology analysis based on degree ranking and the MCC algorithm, AKT1 was prioritized as a candidate target for subsequent molecular docking, molecular dynamics simulation, and mechanistic validation. Because PI3K/AKT signaling was enriched at the pathway level, whereas AKT1 emerged as the prioritized candidate within this axis, the subsequent *in vitro* experiments focused mainly on pathway-level activity, as reflected by the p-PI3K/PI3K and p-AKT/AKT ratios together with pharmacological modulation using SC79 and MK-2206.

#### 3.1.4 Molecular docking validation

Molecular docking analysis predicted a favorable interaction between WGS and AKT1, with WGS occupying the kinase-domain binding pocket of AKT1 (Figure 3C). The docking score was −9.019 kcal/mol, suggesting favorable binding propensity at the computational level. Visualization of the docking pose further showed that WGS was well accommodated within this pocket through spatial complementarity, supporting a plausible ligand-protein interaction mode. These findings provided an initial structural basis for the subsequent molecular dynamics simulation and downstream mechanistic experiments.

#### 3.1.5 Molecular dynamics simulation validation

**Figure 4:**
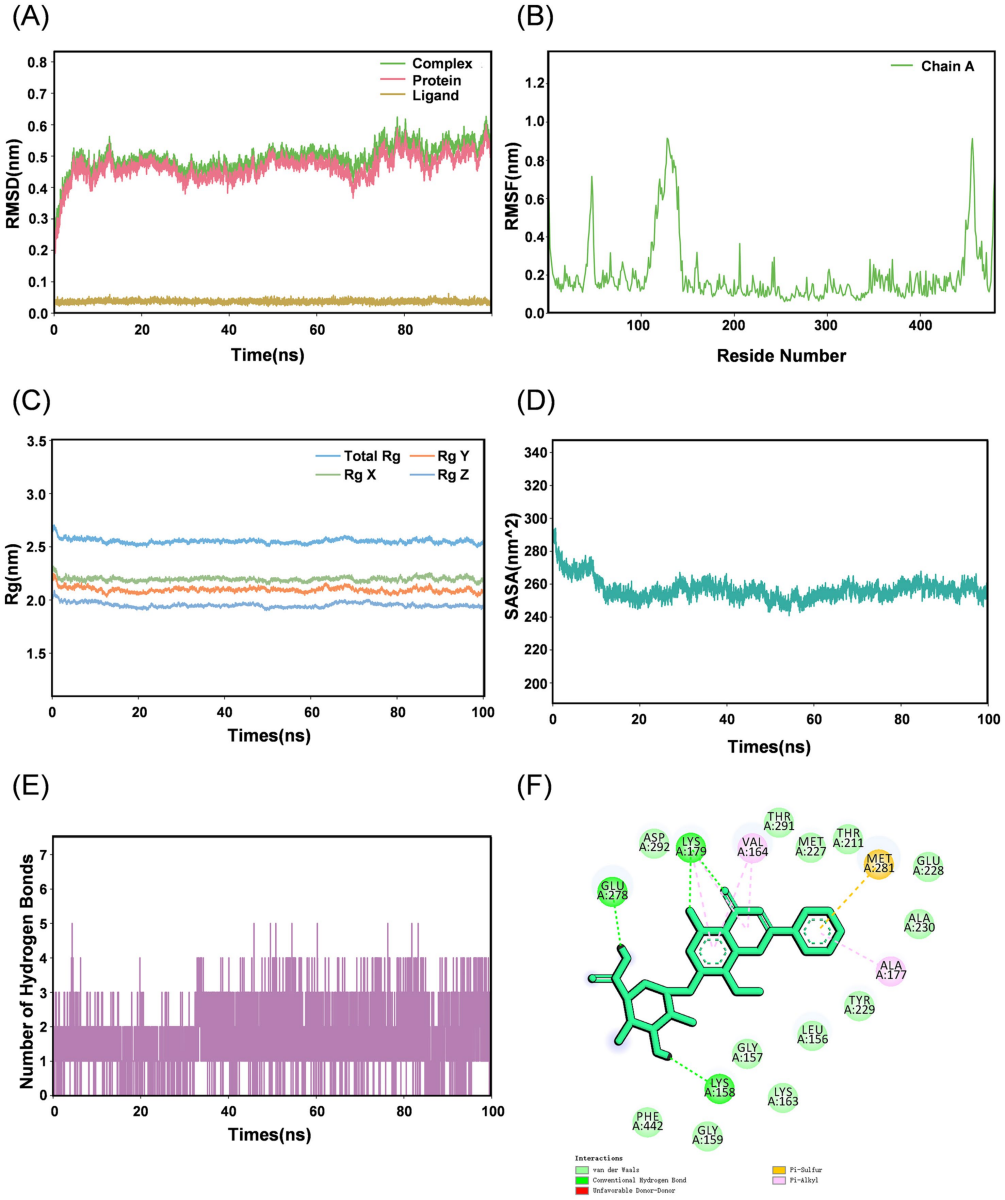
Molecular dynamics simulation analysis of the AKT1-WGS complex. **(A)** Root mean square deviation (RMSD) of the AKT1-WGS complex during the 100 ns simulation. **(B)** Root mean square fluctuation (RMSF) of AKT1 residues in the AKT1-WGS complex. **(C)** Radius of gyration (Rg) of the AKT1-WGS complex during the simulation. **(D)** Solvent-accessible surface area (SASA) of the AKT1-WGS complex during the simulation. **(E)** Number of intermolecular hydrogen bonds formed between WGS and AKT1 throughout the simulation. **(F)** Two-dimensional interaction map of the representative AKT1-WGS binding conformation.

**Figure 5:**
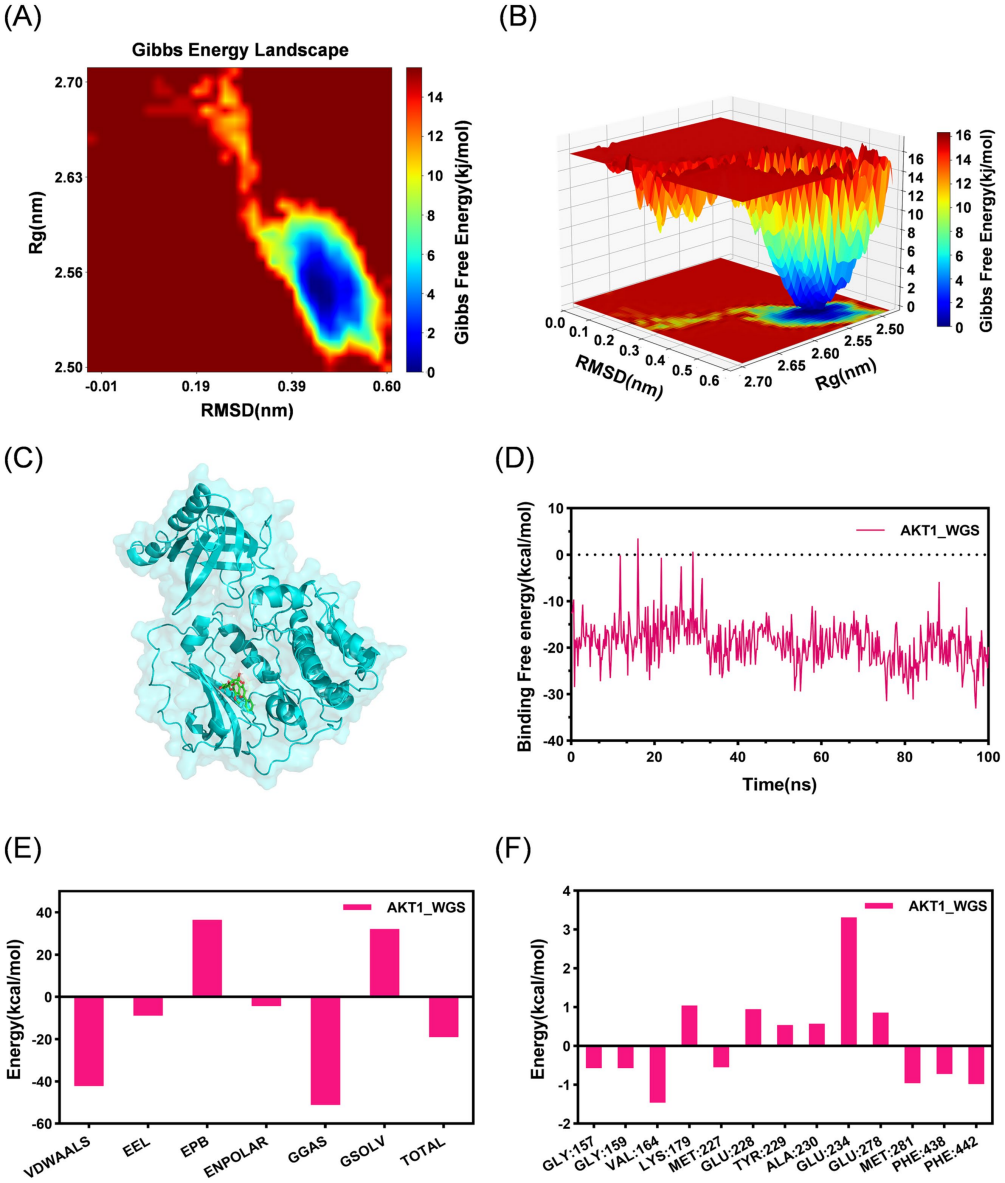
Free energy landscape and binding free energy analysis of the AKT1-WGS complex. **(A)** Two-dimensional free energy landscape of the AKT1-WGS complex based on RMSD and radius of gyration (Rg). **(B)** Three-dimensional free energy landscape of the AKT1-WGS complex. **(C)** Representative low-energy binding conformation extracted from the global minimum basin. **(D)** Binding free energy changes of the AKT1-WGS complex during the 100 ns simulation. **(E)** Total binding free energy and energy component analysis of the AKT1-WGS complex. **(F)** Per-residue free energy contribution to WGS binding to AKT1.

To further characterize the dynamic binding behavior of WGS toward AKT1, the AKT1-WGS complex was subjected to three independent 100 ns molecular dynamics simulations. Three independent simulations showed similar dynamic trends. Representative trajectories are presented in Figures 4, 5, whereas the results of the other two replicate simulations are provided in the Supplementary material. In the representative simulation, RMSD analysis showed that the complex reached a relatively stable state at approximately 20 ns. Although a transient fluctuation was observed around 70 ns, the complex subsequently returned to dynamic equilibrium, and the RMSD values remained generally stable thereafter (Figure 4A). RMSF analysis indicated that regions with relatively high flexibility were mainly located outside the kinase-domain binding pocket (Figure 4B). The Rg and SASA profiles remained generally stable over time, suggesting that WGS binding did not induce marked structural destabilization of AKT1 (Figures 4C,D). Intermolecular hydrogen bonds were maintained during the simulation, supporting persistent ligand-protein interactions (Figure 4E). FEL analysis revealed a distinct low-energy basin, and MM/PBSA analysis yielded a negative total binding free energy, supporting a favorable binding pattern of WGS toward AKT1 (Figure 5). Taken together, these representative results, together with the similar dynamic trends observed across the three independent simulations, support a relatively stable and energetically favorable interaction between WGS and AKT1 over time.

### 3.2 Establishment of the hyperosmotic injury model and dose selection of WGS

#### 3.2.1 Effects of hyperosmotic stress and WGS on cell viability

Cell viability was assessed by CCK-8 assay to determine an appropriate hyperosmotic stress condition and to define the working concentrations of WGS (Figures 1A–C). As the osmolarity of the culture medium increased from 310 to 550 mOsm, the inhibition rate progressively increased, indicating a gradual decline in cell viability in response to hyperosmotic stress (Figure 1A). At 500 mOsm, the inhibition rate increased markedly and approached a half-maximal effect. Accordingly, 500 mOsm was selected as the standardized hyperosmotic condition for the subsequent *in vitro* experiments (42–44).

Under isotonic conditions, 24 h treatment with WGS at concentrations ranging from 25 to 800 μM did not significantly affect cell viability compared with the Control group (Figure 1B), indicating favorable in vitro tolerance within the tested concentration range. The effects of WGS were then evaluated during the intervention phase after hyperosmotic stress induction. Cells were first exposed to 500 mOsm hyperosmotic medium for 24 h and subsequently treated with different concentrations of WGS (25–800 μM). Compared with the HS group, WGS significantly improved cell viability within a defined concentration range. In particular, the 50 μM and 100 μM groups showed significant increases in cell viability (*p* < 0.05), whereas no further improvement was observed at higher concentrations (Figure 1C). Based on these results, 25, 50, and 100 μM were selected as the low-, medium-, and high-dose concentrations for subsequent experiments, with 50 μM chosen for the downstream mechanistic studies.

#### 3.2.2 Effects of WGS on inflammatory mediator levels under hyperosmotic stress

**Figure 6:**
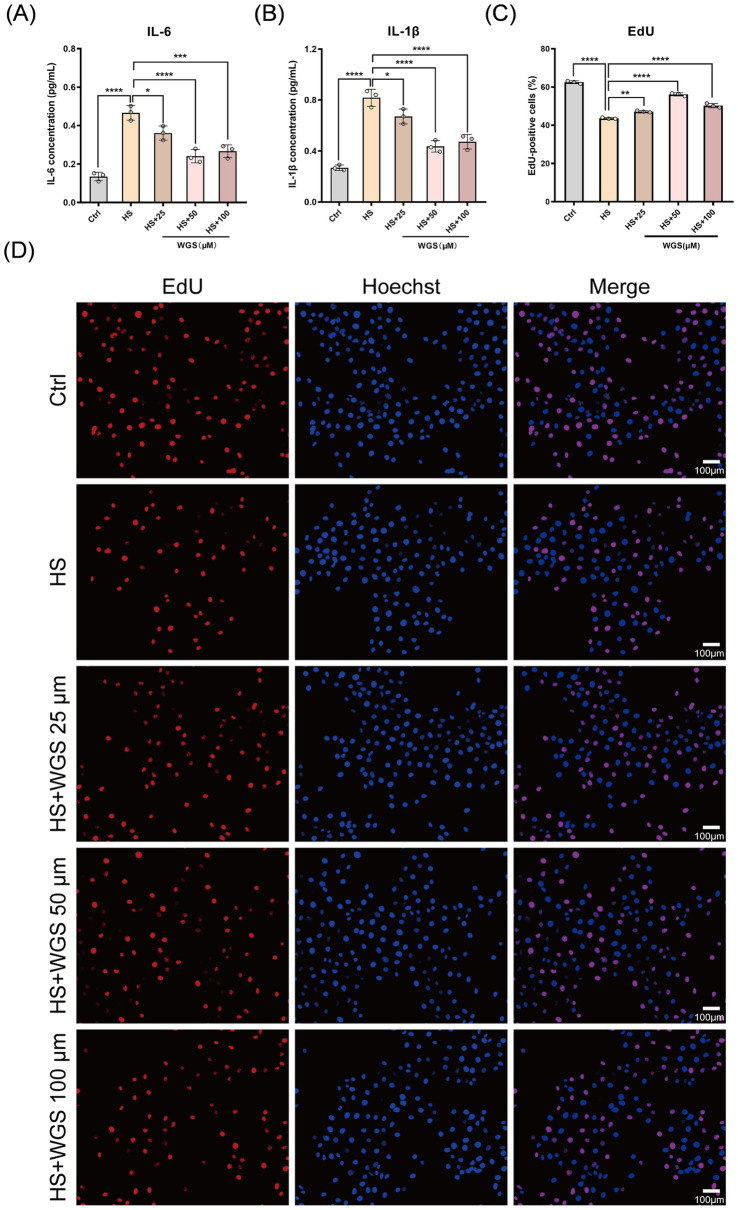
Effects of WGS on inflammatory mediator levels and cell proliferation in HCE-T cells under hyperosmotic stress. **(A)** ELISA measurement of IL-1β levels in culture supernatants. **(B)** ELISA measurement of IL-6 levels in culture supernatants. **(C)** Quantification of EdU-positive cells. **(D)** Representative EdU staining images. EdU-positive cells are shown in red, and nuclei were counterstained with Hoechst 33342 (blue). Merged images are shown in the right column. Data are presented as mean ± standard deviation (SD) from three independent experiments and analyzed by one-way ANOVA followed by Tukey’s multiple-comparisons test. ns, not significant; *p < 0.05, **p < 0.01, ***p < 0.001, ****p < 0.0001.

To assess inflammatory responses, the levels of IL-6 and IL-1β in culture supernatants were measured by ELISA (Figures 6A,B). Compared with the Control group, hyperosmotic stress markedly increased the levels of both cytokines (*p* < 0.0001). WGS treatment at 25, 50, and 100 μM reduced IL-6 and IL-1β levels to varying degrees, with the most pronounced suppression observed at 50 μM (*p* < 0.0001). These findings indicate that WGS attenuates hyperosmotic stress-induced inflammatory mediator production in HCE-T cells.

#### 3.2.3 Effects of WGS on cell proliferation, apoptosis, and AKT phosphorylation

Cell proliferation was evaluated by EdU incorporation assay (Figures 6C,D). Compared with the Control group, the proportion of EdU-positive cells was significantly reduced in the HS group (*p* < 0.0001), indicating impaired proliferative capacity under hyperosmotic stress. WGS treatment at 25, 50, and 100 μM significantly increased the proportion of EdU-positive cells relative to the HS group (*p* < 0.01), with the most prominent effect observed at 50 μM (*p* < 0.0001). These results suggest that WGS partially restores proliferative activity in HCE-T cells under hyperosmotic stress.

**Figure 7:**
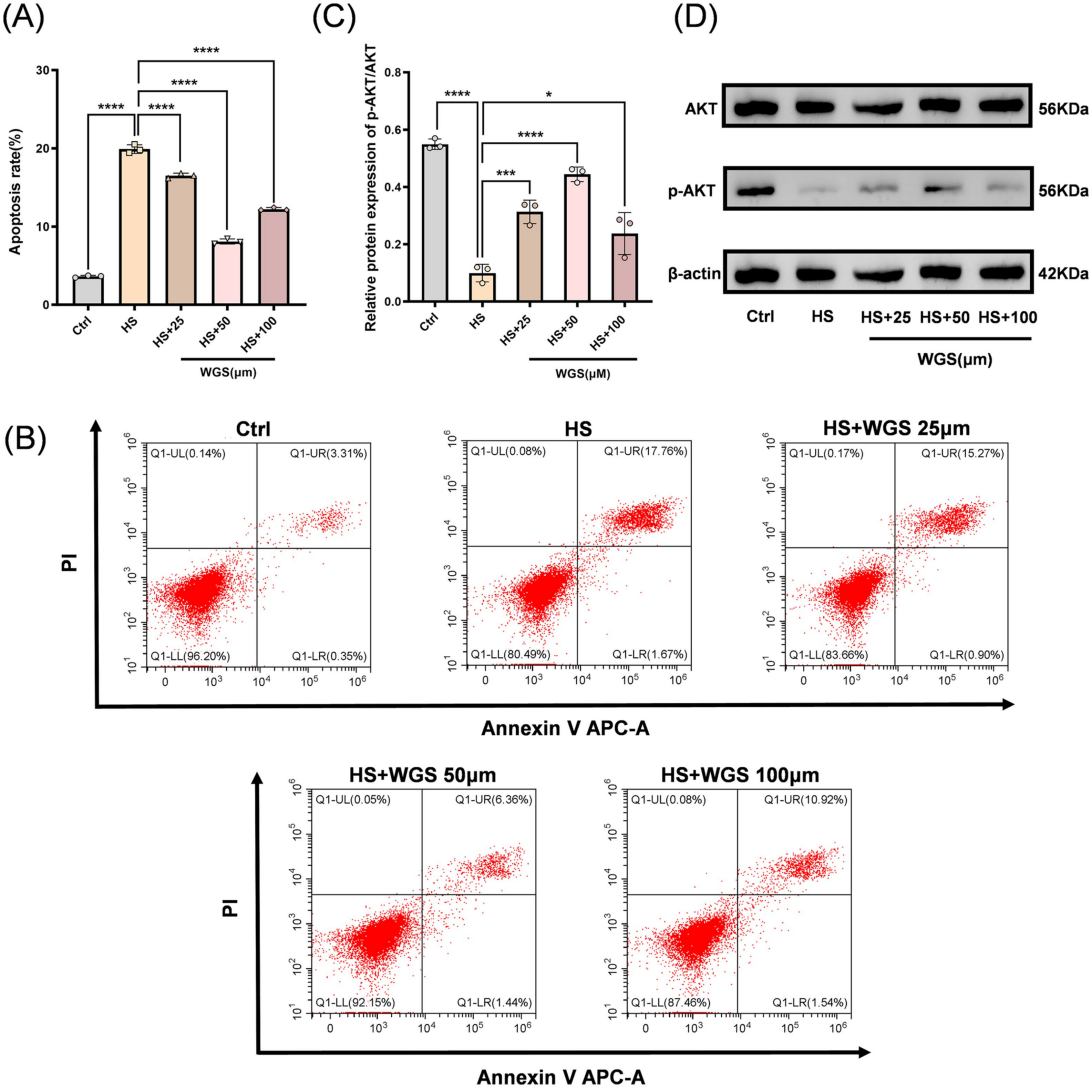
WGS attenuates hyperosmotic stress-induced apoptosis and restores AKT phosphorylation in HCE-T cells. **(A)** Quantification of apoptotic rate determined by Annexin V-APC/PI flow cytometry. **(B)** Representative Annexin V-APC/PI flow cytometry dot plots showing the distribution of viable and apoptotic cells across treatments. **(C)** Densitometric quantification of AKT and p-AKT (Ser473), including the p-AKT/AKT ratio. **(D)** Representative Western blot bands of AKT and p-AKT (Ser473), with β-actin used as the loading control. Data are presented as mean ± standard deviation (SD) from three independent experiments and analyzed by one-way ANOVA followed by Tukey’s multiple-comparisons test. ns, not significant; *p < 0.05, **p < 0.01, ***p < 0.001, ****p < 0.0001.

Apoptosis was further assessed by Annexin V/PI double staining followed by flow cytometry (Figures 7A,B). The apoptotic rate was markedly elevated in the HS group compared with the Control group (*p* < 0.0001). WGS treatment at 25, 50, and 100 μM significantly attenuated hyperosmotic stress-induced apoptosis (*p* < 0.0001), again with the strongest protective effect observed at 50 μM.

Because PI3K/AKT signaling is closely associated with cell survival, AKT activation was further evaluated by Western blot analysis (Figures 7C,D). Compared with the Control group, hyperosmotic stress significantly reduced p-AKT (Ser473) levels and the p-AKT/AKT ratio (*p* < 0.0001). In contrast, WGS treatment at 25, 50, and 100 μM increased p-AKT (Ser473) levels and restored the p-AKT/AKT ratio under HS conditions (*p* < 0.05), with the most prominent recovery observed at 50 μM (*p* < 0.0001).

Taken together, across multiple endpoints, including cell viability, inflammatory mediator levels, proliferation, apoptosis, and AKT phosphorylation, 50 μM WGS consistently exhibited the most favorable protective profile and was therefore selected as the working concentration for the subsequent mechanistic experiments.

### 3.3 PI3K/AKT-dependent protective effects of WGS against hyperosmotic injury

#### 3.3.1 WGS improves cell viability under hyperosmotic stress

The effects of the AKT activator SC79 and the AKT inhibitor MK-2206 on HCE-T cell viability were first assessed by CCK-8 assay to determine appropriate working concentrations for the subsequent mechanistic experiments. Within the tested range, MK-2206 reduced cell viability in a concentration-dependent manner. Therefore, 2.5 μM was selected as the working concentration because it maintained acceptable cellular tolerance (cell viability, 90.47%) (Figure 1D). SC79, used as a positive control for AKT activation, was applied at 10 μM based on CCK-8-derived viability data and previous reports (cell viability, 97.69%) (Figures 1E,F).

**Figure 8:**
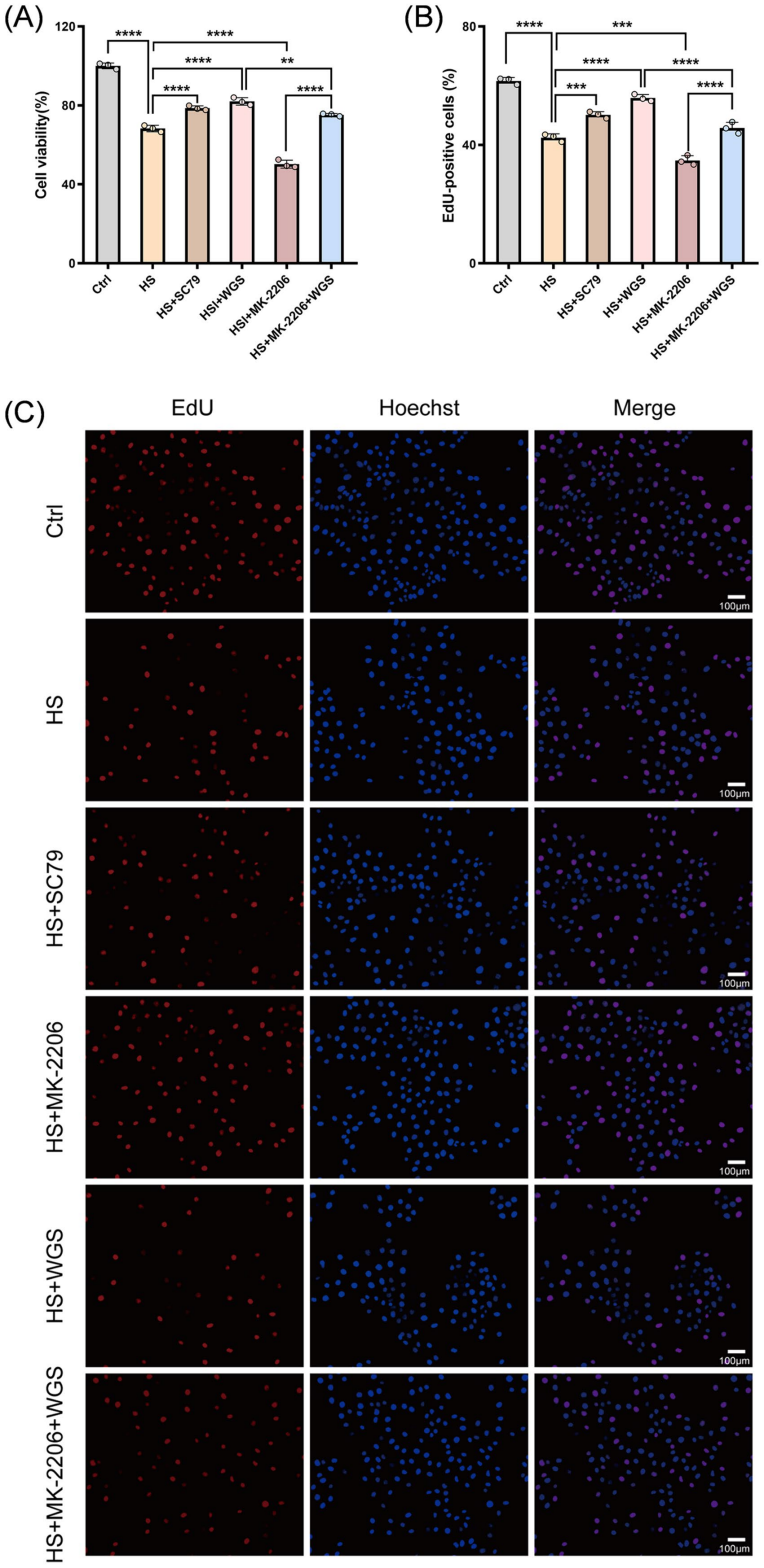
Effects of WGS on HCE-T cell viability and proliferation under hyperosmotic stress under AKT activation or inhibition. **(A)** CCK-8 assay showing cell viability across the indicated treatment groups. **(B)** Quantification of EdU-positive cells. **(C)** Representative EdU staining images. EdU-positive cells are shown in red, and nuclei were counterstained with Hoechst 33342 (blue). Merged images are shown in the right column. Data are presented as mean ± standard deviation (SD) from three independent experiments and analyzed by one-way ANOVA followed by Tukey’s multiple-comparisons test. ns, not significant; *p < 0.05, **p < 0.01, ***p < 0.001, ****p < 0.0001.

In the six-group mechanistic comparison, exposure to hyperosmotic stress for 24 h significantly reduced cell viability relative to the Control group (*p* < 0.0001). Compared with the HS group, MK-2206 further decreased cell viability (*p* < 0.0001), whereas SC79 markedly increased viability (*p* < 0.0001). WGS treatment (50 μM) significantly improved cell viability under HS conditions (*p* < 0.0001). Co-treatment with MK-2206 partially attenuated the protective effect of WGS, as reflected by lower viability than that in the WGS group (*p* < 0.01), although viability remained significantly higher than that in the MK-2206 group (*p* < 0.0001) (Figure 8A).

#### 3.3.2 WGS restores cell proliferation under hyperosmotic stress

Cell proliferation was evaluated by EdU incorporation assay (Figures 8B,C). Compared with the Control group, the proportion of EdU-positive cells was markedly reduced in the HS group (*p* < 0.0001), indicating impaired proliferative capacity under hyperosmotic stress. Relative to the HS group, MK-2206 further suppressed cell proliferation (*p* < 0.001), whereas SC79 significantly enhanced proliferative activity (*p* < 0.001). WGS treatment produced a pronounced increase in the proportion of EdU-positive cells compared with the HS group (*p* < 0.0001). Under combined treatment with MK-2206 and WGS, proliferation was lower than that observed in the WGS group (*p* < 0.0001) but remained higher than that in the MK-2206 group (*p* < 0.0001).

#### 3.3.3 WGS reduces hyperosmotic stress-induced apoptosis

**Figure 9:**
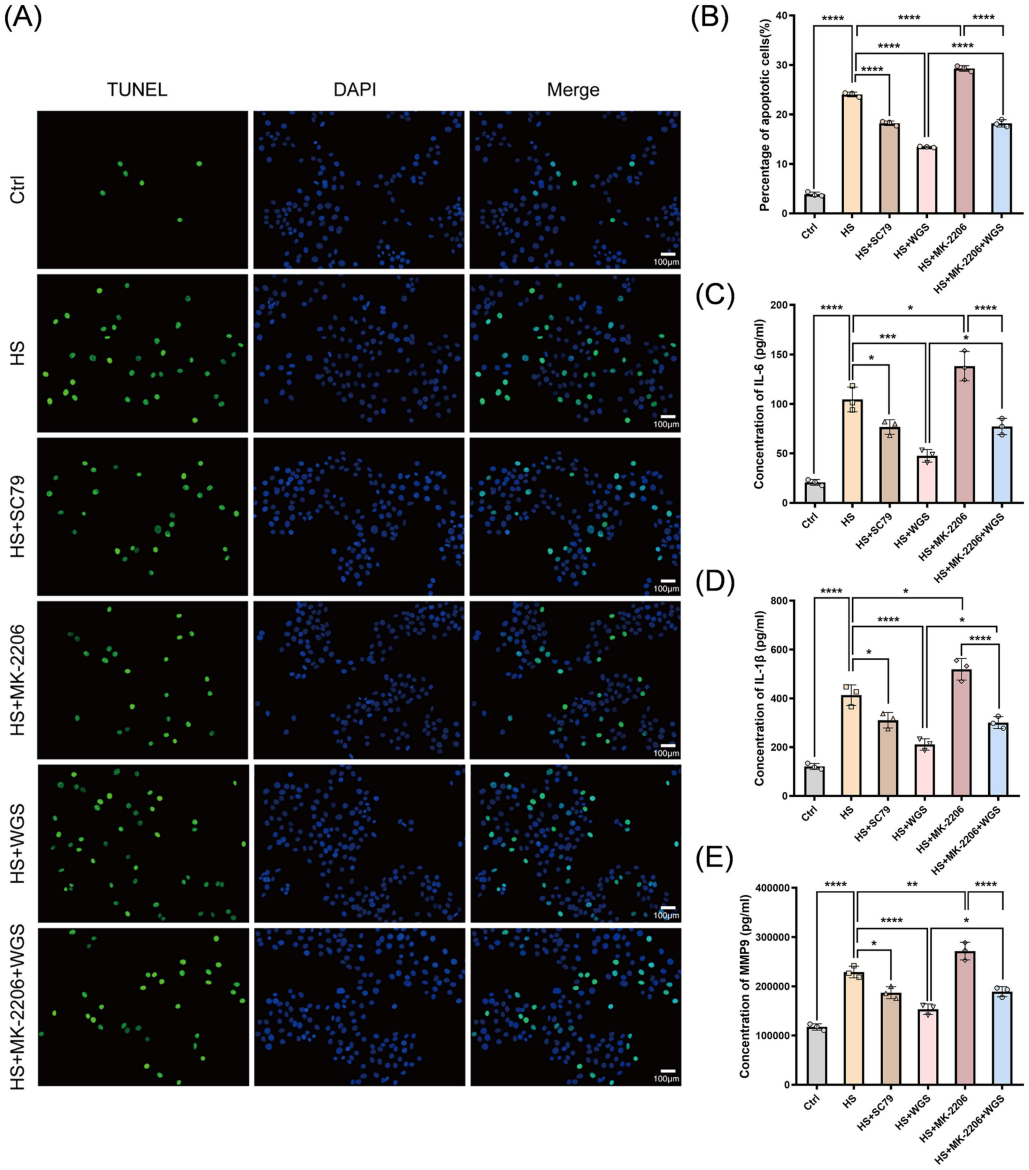
Effects of WGS on apoptosis and inflammatory mediator levels in HCE-T cells under hyperosmotic stress with AKT activation or inhibition. **(A)** Representative TUNEL staining images. TUNEL-positive nuclei are shown in green, and nuclei were counterstained with DAPI (blue). Merged images are shown in the right column. **(B)** Quantification of TUNEL-positive cells. **(C–E)** ELISA measurement of IL-6 **(C)**, IL-1β **(D)**, and MMP-9, **(E)** levels in culture supernatants. Data are presented as mean ± standard deviation (SD) from three independent experiments and analyzed by one-way ANOVA followed by Tukey’s multiple-comparisons test. ns, not significant; *p < 0.05, **p < 0.01, ***p < 0.001, ****p < 0.0001.

Apoptosis was assessed by TUNEL staining (Figures 9A,B). Compared with the Control group, the proportion of TUNEL-positive cells was significantly increased in the HS group (*p* < 0.0001), indicating enhanced apoptosis under hyperosmotic stress. Relative to the HS group, MK-2206 further increased the percentage of TUNEL-positive cells (*p* < 0.0001), whereas SC79 markedly reduced apoptosis (*p* < 0.0001). WGS treatment significantly decreased the proportion of TUNEL-positive cells compared with the HS group (*p* < 0.0001). Under combined treatment with MK-2206 and WGS, apoptosis was higher than that observed in the WGS group (*p* < 0.0001) but lower than that in the MK-2206 group (*p* < 0.0001).

#### 3.3.4 WGS suppresses hyperosmotic stress-induced inflammatory mediator production

The levels of IL-6, IL-1β, and MMP-9 in culture supernatants were quantified by ELISA (Figures 9C–E). Compared with the Control group, hyperosmotic stress significantly increased the levels of IL-1β, IL-6, and MMP-9 (*p* < 0.0001), indicating enhanced inflammatory mediator production. Relative to the HS group, MK-2206 further increased the levels of these mediators (*p* < 0.05), whereas SC79 reduced them (*p* < 0.05). WGS treatment significantly decreased IL-1β, IL-6, and MMP-9 levels compared with the HS group (*p* < 0.001). Under combined treatment with MK-2206 and WGS, cytokine and MMP-9 levels were higher than those in the WGS group (*p* < 0.05) but lower than those in the MK-2206 group (*p* < 0.0001).

#### 3.3.5 WGS restores PI3K/AKT phosphorylation under hyperosmotic stress

**Figure 10:**
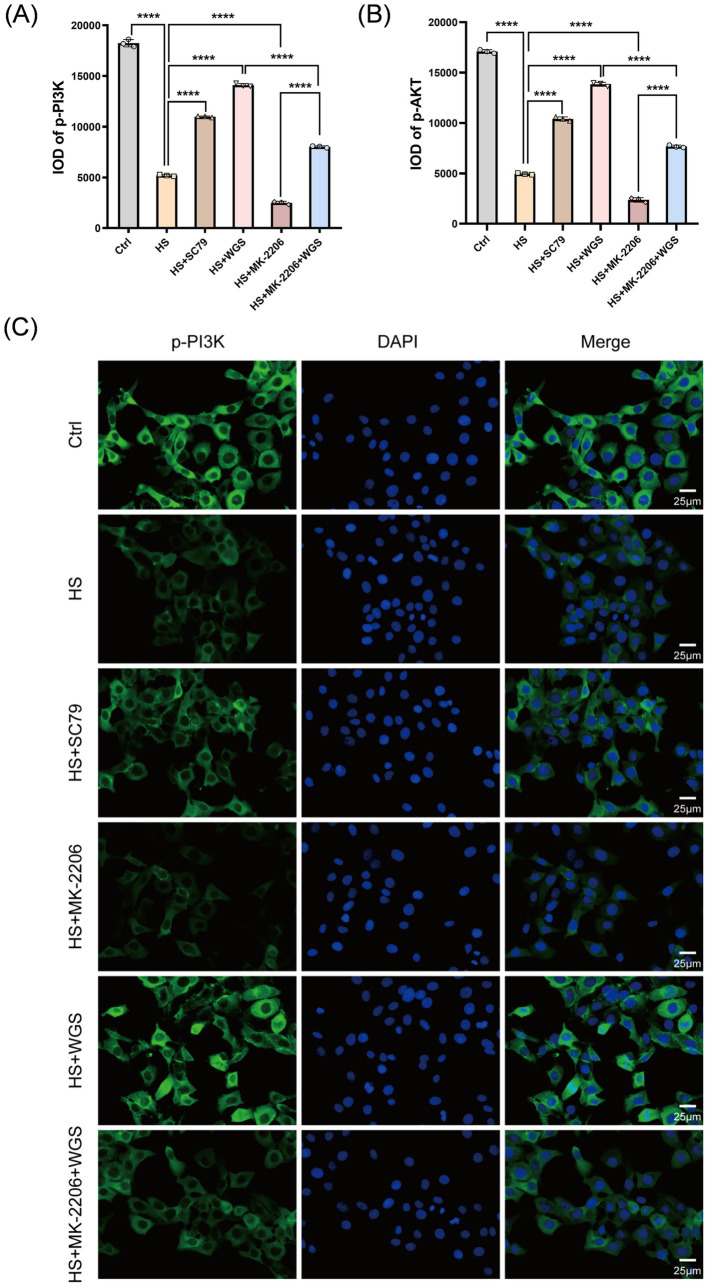
Immunofluorescence analysis of PI3K/AKT pathway activation in HCE-T cells under hyperosmotic stress with AKT activation or inhibition. **(A)** Quantification of p-PI3K immunofluorescence intensity, presented as integrated optical density (IOD). **(B)** Quantification of p-AKT (Ser473) immunofluorescence intensity, presented as IOD. **(C)** Representative immunofluorescence images of p-PI3K staining (green) with DAPI nuclear counterstaining (blue). Merged images are shown in the right column. Data are presented as mean ± standard deviation (SD) from three independent experiments and analyzed by one-way ANOVA followed by Tukey’s multiple-comparisons test. ns, not significant; *p < 0.05, **p < 0.01, ***p < 0.001, ****p < 0.0001.

**Figure 11:**
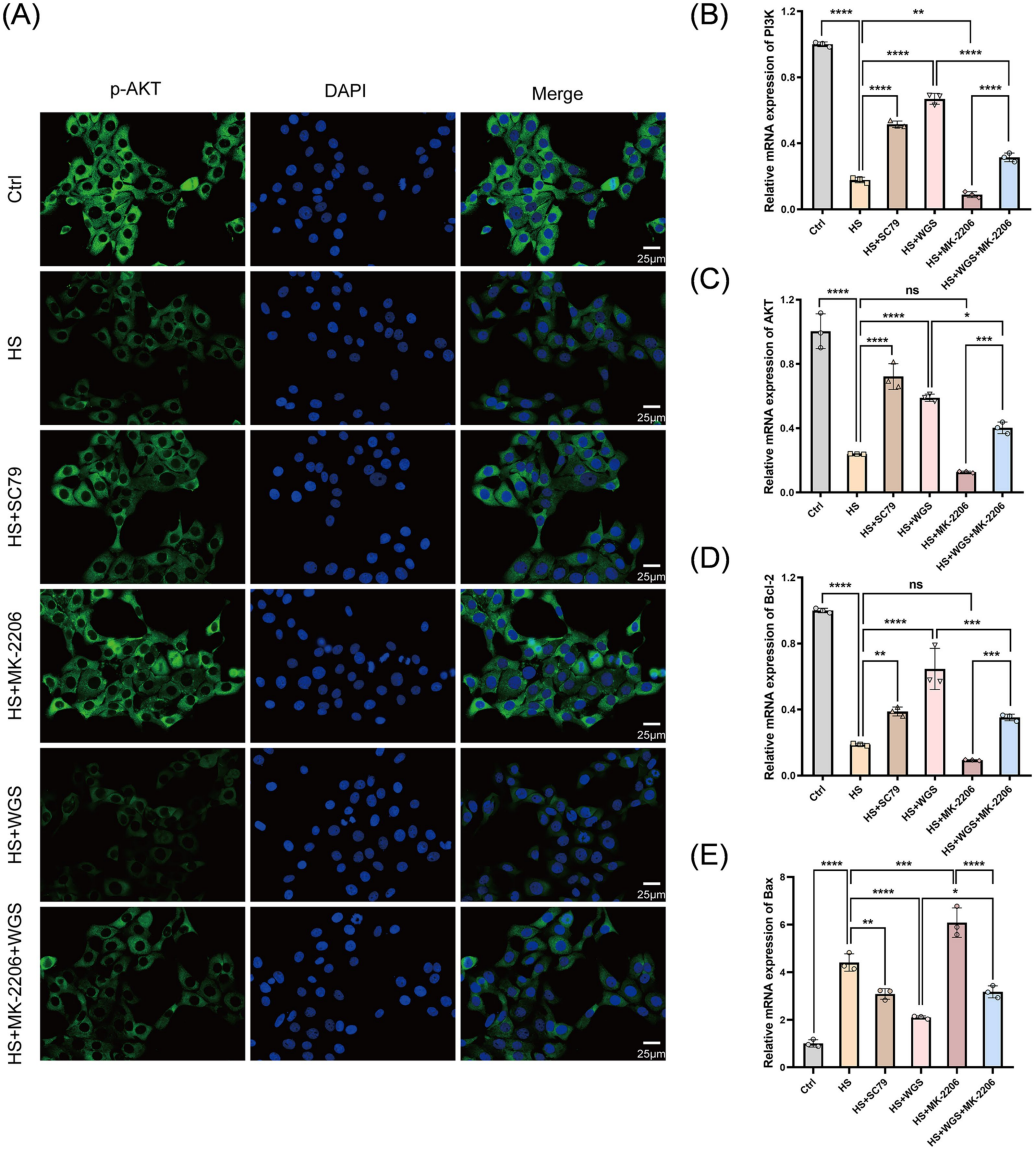
Effects of WGS on p-AKT immunofluorescence and the mRNA expression of PI3K/AKT pathway- and apoptosis-related genes in HCE-T cells under hyperosmotic stress with AKT activation or inhibition. **(A)** Representative immunofluorescence images of p-AKT (Ser473) staining (green) with DAPI nuclear counterstaining (blue). Merged images are shown in the right column. **(B–E)** RT-qPCR analysis of PI3K **(B)**, AKT **(C)**, Bcl-2 **(D)**, and Bax **(E)** mRNA expression levels. Data are presented as mean ± standard deviation (SD) from three independent biological samples per group and analyzed by one-way ANOVA followed by Tukey’s multiple-comparisons test. ns, not significant; *p < 0.05, **p < 0.01, ***p < 0.001, ****p < 0.0001.

Immunofluorescence staining was performed to detect p-PI3K and p-AKT (Ser473) in each group (Figures 10A–C, 11A). Compared with the Control group, the HS group exhibited significantly reduced p-PI3K and p-AKT fluorescence signals (*p* < 0.0001), consistent with the phosphorylation changes observed by Western blot analysis. Relative to the HS group, MK-2206 further reduced p-PI3K and p-AKT fluorescence signals (*p* < 0.0001), whereas SC79 significantly enhanced these signals (*p* < 0.0001). Compared with the HS group, WGS treatment markedly increased p-PI3K and p-AKT fluorescence signals (*p* < 0.0001). Under combined treatment with MK-2206 and WGS, p-PI3K and p-AKT fluorescence signals were lower than those in the WGS group (*p* < 0.0001) but remained higher than those in the MK-2206 group (*p* < 0.0001) (Figures 10A,B).

#### 3.3.6 WGS regulates apoptosis-related mRNA expression through PI3K/AKT signaling

The mRNA expression levels of PI3K, AKT, Bax, and Bcl-2 were quantified by RT-qPCR (Figures 11B–E). Compared with the Control group, the HS group exhibited significantly reduced mRNA levels of PI3K, AKT, and Bcl-2 (*p* < 0.0001), together with a marked increase in Bax mRNA expression (*p* < 0.0001), reflecting a pro-apoptotic transcriptional profile. Relative to the HS group, MK-2206 further downregulated PI3K mRNA expression (*p* < 0.01) while increasing Bax mRNA expression (*p* < 0.001), whereas SC79 produced the opposite pattern, with increased PI3K, AKT, and Bcl-2 mRNA levels and reduced Bax mRNA expression (*p* < 0.01).

Compared with the HS group, WGS treatment significantly increased PI3K, AKT, and Bcl-2 mRNA levels and reduced Bax mRNA expression (*p* < 0.0001). Under combined treatment with MK-2206 and WGS, PI3K, AKT, and Bcl-2 mRNA levels were lower than those in the WGS group (*p* < 0.05), whereas Bax mRNA expression was higher than that in the WGS group (*p* < 0.05).

#### 3.3.7 Effects of WGS on PI3K/AKT signaling and downstream apoptosis- and inflammation-related proteins

**Figure 12:**
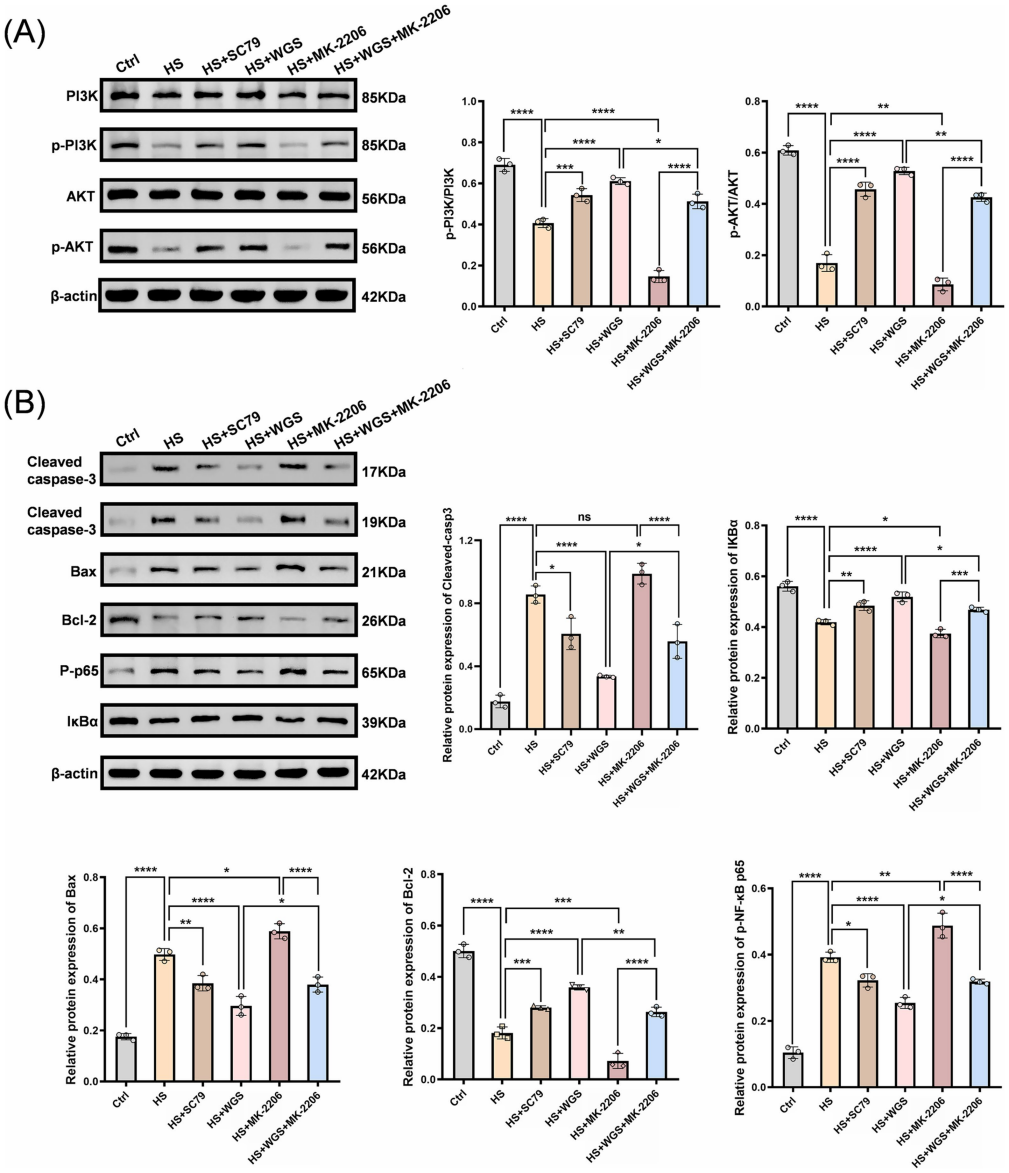
Western blot analysis of PI3K/AKT signaling and downstream apoptosis- and inflammation-related proteins in HCE-T cells under hyperosmotic stress with AKT activation or inhibition. **(A)** Representative immunoblots and densitometric quantification of PI3K/AKT pathway proteins, including PI3K, p-PI3K, AKT, and p-AKT (Ser473). The p-PI3K/PI3K and p-AKT/AKT ratios are shown. **(B)** Representative immunoblots and densitometric quantification of cleaved caspase-3, IκBα, Bax, Bcl-2, and p-NF-κB p65. β-actin was used as the loading control. Data are presented as mean ± standard deviation (SD) from three independent experiments and analyzed by one-way ANOVA followed by Tukey’s multiple-comparisons test. ns, not significant; *p < 0.05, **p < 0.01, ***p < 0.001, ****p < 0.0001.

Western blot analysis was performed to assess PI3K/AKT signaling and downstream apoptosis- and inflammation-related protein changes (Figures 12A,B). Compared with the Control group, hyperosmotic stress significantly decreased the p-PI3K/PI3K and p-AKT/AKT ratios, together with reduced IκBα expression (*p* < 0.0001). In parallel, Bax expression, cleaved caspase-3 levels (calculated as the sum of the p17 and p19 bands), and p-NF-κB p65 levels were significantly increased (*p* < 0.0001), indicating suppression of PI3K/AKT signaling accompanied by activation of apoptosis- and inflammation-related responses.

Pharmacological modulation of AKT further supported these changes. Relative to the HS group, MK-2206 further reduced the p-PI3K/PI3K and p-AKT/AKT ratios as well as IκBα expression, while further increasing Bax expression, cleaved caspase-3 levels, and p-NF-κB p65 levels (*p* < 0.0001). In contrast, SC79 largely reversed these changes, as reflected by restoration of the p-PI3K/PI3K and p-AKT/AKT ratios and IκBα expression, together with reductions in Bax expression, cleaved caspase-3 levels, and p-NF-κB p65 levels (*p* < 0.0001).

WGS treatment markedly reversed the hyperosmotic stress-induced protein changes, as shown by restoration of the p-PI3K/PI3K and p-AKT/AKT ratios and IκBα expression, together with reductions in Bax expression, cleaved caspase-3 levels, and p-NF-κB p65 levels (*p* < 0.0001). Co-treatment with MK-2206 partially attenuated the effects of WGS, as reflected by lower p-PI3K/PI3K and p-AKT/AKT ratios and lower IκBα expression than in the WGS group, together with higher Bax expression, cleaved caspase-3 levels, and p-NF-κB p65 levels. Nevertheless, compared with MK-2206 alone, the MK-2206 + WGS group still showed partial restoration of PI3K/AKT signaling and partial attenuation of apoptosis- and inflammation-related protein changes (*p* < 0.0001). Collectively, these results indicate that the protective effects of WGS against hyperosmotic stress are associated, at least in part, with restoration of PI3K/AKT signaling and modulation of downstream apoptosis- and inflammation-related proteins.

#### 3.3.8 CETSA supports target engagement of WGS with AKT1 in HCE-T cells

Network pharmacology, molecular docking, and molecular dynamics simulation consistently suggested AKT1 as a candidate target of WGS. In parallel, the results above showed that WGS restored PI3K/AKT signaling under hyperosmotic stress in HCE-T cells. We therefore examined whether WGS could affect AKT1 thermal stability at the cellular level using CETSA.

**Figure 13:**
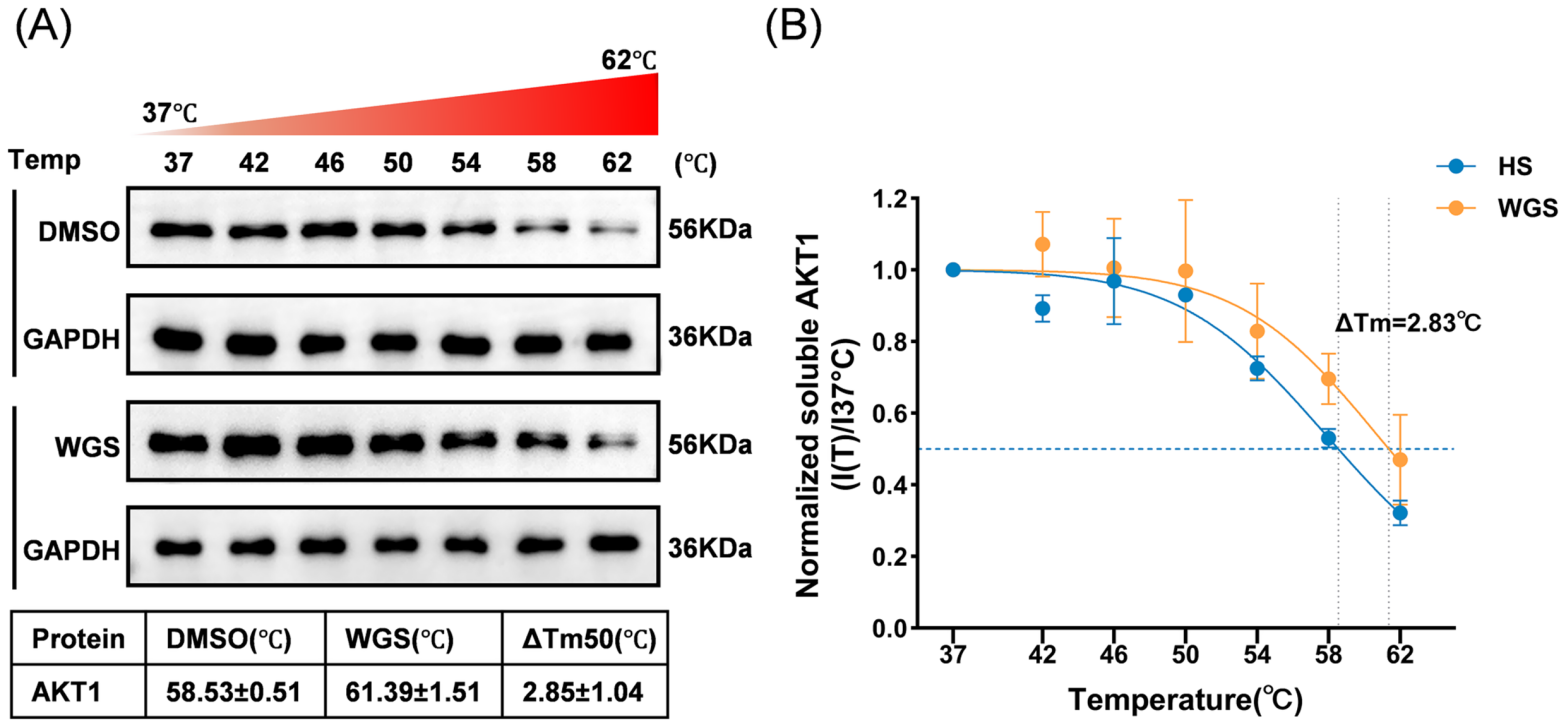
CETSA analysis of AKT1 following WGS treatment in HCE-T cells. **(A)** Representative Western blot images showing soluble AKT1 levels in HCE-T cells treated with DMSO or WGS after heating at the indicated temperatures. GAPDH was used as the loading control. **(B)** Melting curves of soluble AKT1 normalized to the 37 °C sample. WGS treatment shifted the thermal denaturation curve of AKT1 to the right and increased the apparent melting temperature (Tm), indicating enhanced thermal stability of AKT1. Data are presented as mean ± standard deviation (SD) from three independent experiments.

As shown in Figure 13A, the soluble AKT1 signal gradually decreased as temperature increased in both the DMSO and WGS groups, consistent with heat-induced protein denaturation. Notably, compared with the DMSO group, more soluble AKT1 was retained in the WGS group at higher temperatures, particularly at 58 °C and 62 °C, suggesting that WGS enhanced the thermal stability of AKT1 in cells.

This observation was further supported by melting-curve analysis. As shown in Figure 13B, the thermal denaturation curve of AKT1 in the WGS group was shifted to the right relative to that in the DMSO group. Quantitative fitting showed that the apparent melting temperature of AKT1 increased from 58.53 ± 0.51 °C in the DMSO group to 61.39 ± 1.51 °C in the WGS group, with a ΔTm of 2.85 ± 1.04 °C. Taken together, these findings indicate that WGS increases the thermal stability of AKT1 in HCE-T cells and provide additional evidence supporting AKT1 as a cellular target of WGS.

## 4 Discussion

Dry eye is a multifactorial ocular surface disorder characterized by tear film instability, hyperosmolarity, chronic inflammation, and progressive disruption of epithelial homeostasis, ultimately leading to impairment of ocular surface barrier function and visual quality (51). Owing to its high prevalence and persistent impact on daily function and quality of life, dry eye has become an important challenge in the long-term management of ocular surface disease (52, 51). Among the recognized pathogenic drivers, tear hyperosmolarity is widely regarded as a central initiating and perpetuating factor because it reflects tear film disequilibrium and directly activates inflammation- and apoptosis-related signaling in corneal epithelial cells (53). Based on this rationale, we used 500 mOsm hyperosmotic medium to establish an *in vitro* dry eye-related stress model, which is consistent with previously reported experimental settings (44).

Hyperosmotic stress is known to amplify epithelial injury by activating inflammatory cascades and apoptosis-related programs. Earlier studies have shown that, in human corneal epithelial cells, increasing medium osmolarity is accompanied by increased MMP-9 expression and secretion, suggesting that hyperosmotic stress itself is sufficient to trigger stress-responsive pathways that aggravate epithelial damage (54). Once this response is sustained, epithelial injury and impaired repair capacity may reinforce each other and contribute to the chronic pathological cycle of dry eye (55). Against this background, identifying compounds that can simultaneously attenuate inflammation, limit apoptosis, and preserve epithelial function is of particular interest.

The WGS is a flavonoid glycoside derived from Scutellaria baicalensis and has been reported to exert anti-inflammatory and cytoprotective effects through modulation of multiple intracellular signaling pathways involved in cell survival and stress responses (56). In inflammation and injury models, WGS reduces pro-inflammatory cytokine production and regulates downstream gene transcription through the NF-κB signaling pathway, thereby contributing to cellular homeostasis and tissue repair (16, 17, 57). WGS has also been shown to reduce oxidative stress and decrease inflammatory mediators such as IL-1β and IL-6 (15). Collectively, these findings provide a pharmacological basis for evaluating WGS in hyperosmolarity-induced ocular surface inflammation. In the present study, we used network pharmacology to explore the potential mechanisms underlying the effects of WGS in dry eye and further validated its protective effects under hyperosmotic stress in HCE-T cells.

At the systems level, network pharmacology suggested that WGS may act through multiple targets and pathways relevant to dry eye. The overlapping target set was mainly enriched in biological processes associated with inflammatory regulation, apoptosis, and cellular stress responses, whereas KEGG pathway analysis highlighted PI3K/AKT signaling as a prominent pathway. These findings are biologically plausible, as previous studies have linked PI3K/AKT activity to corneal epithelial survival, inflammatory regulation, and ocular surface repair under stress conditions (58, 59, 60). Within this pathway-oriented context, AKT1 was prioritized as a candidate target by topological analysis, providing a structural focus for subsequent docking, molecular dynamics simulation, and CETSA. However, because the enrichment result primarily supported pathway-level involvement rather than isoform-specific biological output, the subsequent cell-based experiments were designed mainly to evaluate PI3K/AKT pathway activity using phosphorylation-based readouts and pharmacological modulation, rather than isoform-specific interrogation of AKT1 alone (45–47).

Our cellular experiments supported a beneficial effect of WGS during the intervention phase after hyperosmotic stress induction. WGS showed no obvious cytotoxicity within the tested concentration range under isotonic conditions and improved cell viability after hyperosmotic stress induction, with 50 μM showing the most favorable overall profile. This concentration-dependent trend was further supported by EdU data, which showed partial restoration of proliferative capacity in HCE-T cells after hyperosmotic exposure. These findings suggest that WGS may facilitate recovery from hyperosmotic stress-induced epithelial injury.

Inflammation and apoptosis are central pathological events in dry eye progression and are closely linked to epithelial barrier dysfunction and defective tissue repair (61). Under hyperosmotic conditions, innate inflammatory signaling is activated at the ocular surface, leading to increased release of pro-inflammatory mediators and enhanced epithelial apoptosis (62, 63). In the present study, WGS significantly reduced IL-1β, IL-6, and MMP-9 levels, decreased TUNEL positivity and apoptotic rate, and partially restored proliferative activity. Together, these findings indicate that WGS attenuates inflammatory and apoptotic injury in HCE-T cells during the intervention phase after hyperosmotic stress induction.

This anti-injury effect was further supported at the transcriptional and protein levels. WGS increased PI3K, AKT, and Bcl-2 mRNA expression, while reducing Bax mRNA expression, indicating a shift away from a pro-apoptotic transcriptional profile. Consistent with this, Western blot analysis showed that WGS restored the p-PI3K/PI3K and p-AKT/AKT ratios, reduced Bax expression and cleaved caspase-3 levels, and was accompanied by reduced p-NF-κB p65 levels together with restoration of IκBα expression. These results suggest that WGS-mediated protection involves coordinated suppression of apoptosis- and inflammation-related responses downstream of PI3K/AKT signaling, rather than a single isolated endpoint.

The PI3K/AKT pathway is a well-recognized regulator of epithelial survival, stress adaptation, and inflammatory control (64, 65). Previous studies in corneal epithelial cells have shown that activation of AKT enhances tolerance to hyperosmotic and inflammatory stress, whereas impaired PI3K/AKT signaling is associated with increased apoptosis and delayed epithelial recovery (66). In our model, hyperosmotic stress markedly suppressed PI3K/AKT phosphorylation, whereas WGS restored both p-PI3K and p-AKT levels. This association was further strengthened by pharmacological intervention: SC79 enhanced the protective phenotype, whereas MK-2206 aggravated injury and partially attenuated the effects of WGS. Taken together, these data support the conclusion that PI3K/AKT signaling plays a functional role in the epithelial protection mediated by WGS.

The *in silico* structural analyses further strengthened the biological findings. Molecular docking suggested a favorable interaction between WGS and AKT1 within the kinase-domain binding pocket, and molecular dynamics simulation showed that the AKT1-WGS complex remained conformationally stable throughout the 100 ns trajectory. RMSD, RMSF, Rg, and SASA analyses collectively supported structural stability, while FEL analysis indicated a thermodynamically favorable conformational basin. In addition, MM/PBSA analysis showed a persistently negative binding free energy, and residue-wise decomposition identified several residues, including GLY157, GLY159, VAL164, GLU278, MET281, PHE438, and PHE442, as important contributors to ligand binding. These results do not by themselves establish direct biochemical binding, but they provide coherent structural support for the plausibility of AKT1 as a candidate target of WGS and strengthen the interpretation of the pathway-level cellular data.

Importantly, CETSA provided additional cellular-level evidence supporting target engagement. Compared with the DMSO group, WGS increased the thermal stability of AKT1 in HCE-T cells and shifted the melting curve to the right, with an apparent increase in Tm. This finding extends the docking and molecular dynamics results by suggesting that WGS can influence AKT1 stability in the cellular context. Therefore, the overall evidence chain in this study is internally consistent: network pharmacology prioritized AKT1 within a PI3K/AKT-centered pathway framework, docking and molecular dynamics supported structural feasibility, CETSA supported cellular target engagement, and cell-based experiments demonstrated restoration of PI3K/AKT signaling together with attenuation of downstream inflammatory and apoptotic injury.

Several limitations should nevertheless be acknowledged. First, the hyperosmotic HCE-T cell model reproduces key stress features of dry eye but cannot fully capture the complexity of the ocular surface microenvironment, including immune, neural, and tear film-related interactions (67). In addition, because the intervention phase began after replacement of the hyperosmotic medium with isotonic medium, some degree of spontaneous recovery cannot be excluded. Similar post-stress intervention designs have been reported in corneal epithelial studies, and recovery after hyperosmotic exposure may remain incomplete even after return to normal medium (48). The present findings should therefore be interpreted as effects observed after established stress injury rather than protection under continuously sustained hyperosmotic conditions. Second, although SC79 and MK-2206 are useful tools for pathway-level interrogation, their effective concentrations and cellular responses may vary across experimental systems (68). Third, while the combined evidence supports AKT1 as a candidate cellular target of WGS, the *in vitro* validation in this study primarily reflects pathway-level PI3K/AKT activity rather than isoform-specific functional dissection of AKT1 alone. More selective target perturbation strategies, together with appropriate animal models of dry eye, are needed to further refine the mechanistic interpretation and evaluate the *in vivo* relevance of WGS.

In summary, WGS attenuated hyperosmotic stress-induced corneal epithelial injury in HCE-T cells, at least in part, by restoring PI3K/AKT signaling and suppressing downstream inflammatory and apoptotic responses. Evidence from network pharmacology, molecular docking, molecular dynamics simulation, CETSA, and in vitro experiments supports AKT1 as a candidate target of WGS and provides a rationale for further evaluation in appropriate animal models of dry eye.

## 5 Conclusion

In conclusion, WGS alleviated hyperosmotic stress-induced injury in HCE-T cells, as reflected by improved cell viability and proliferation, reduced apoptosis, and decreased levels of inflammatory mediators, including IL-1β, IL-6, and MMP-9. These protective effects were associated with restoration of PI3K/AKT signaling. Integrated evidence from network pharmacology, molecular docking, molecular dynamics simulation, CETSA, and in vitro experiments supports AKT1 as a candidate target of WGS. Collectively, these findings suggest that WGS may be a candidate compound for mitigating hyperosmotic stress-related corneal epithelial injury and deserves further validation in appropriate animal models of dry eye.

## Data Availability

The raw data supporting the conclusions of this article will be made available by the authors, without undue reservation.
