# Supplementary figures and images for: Wogonoside alleviates hyperosmotic stress-induced inflammation and apoptosis in human corneal epithelial cells via PI3K/AKT signaling
Source: Front Med (Lausanne). 2026 May 4;13:1828021. doi: 10.3389/fmed.2026.1828021 (PMC13214516; doi:10.3389/fmed.2026.1828021)

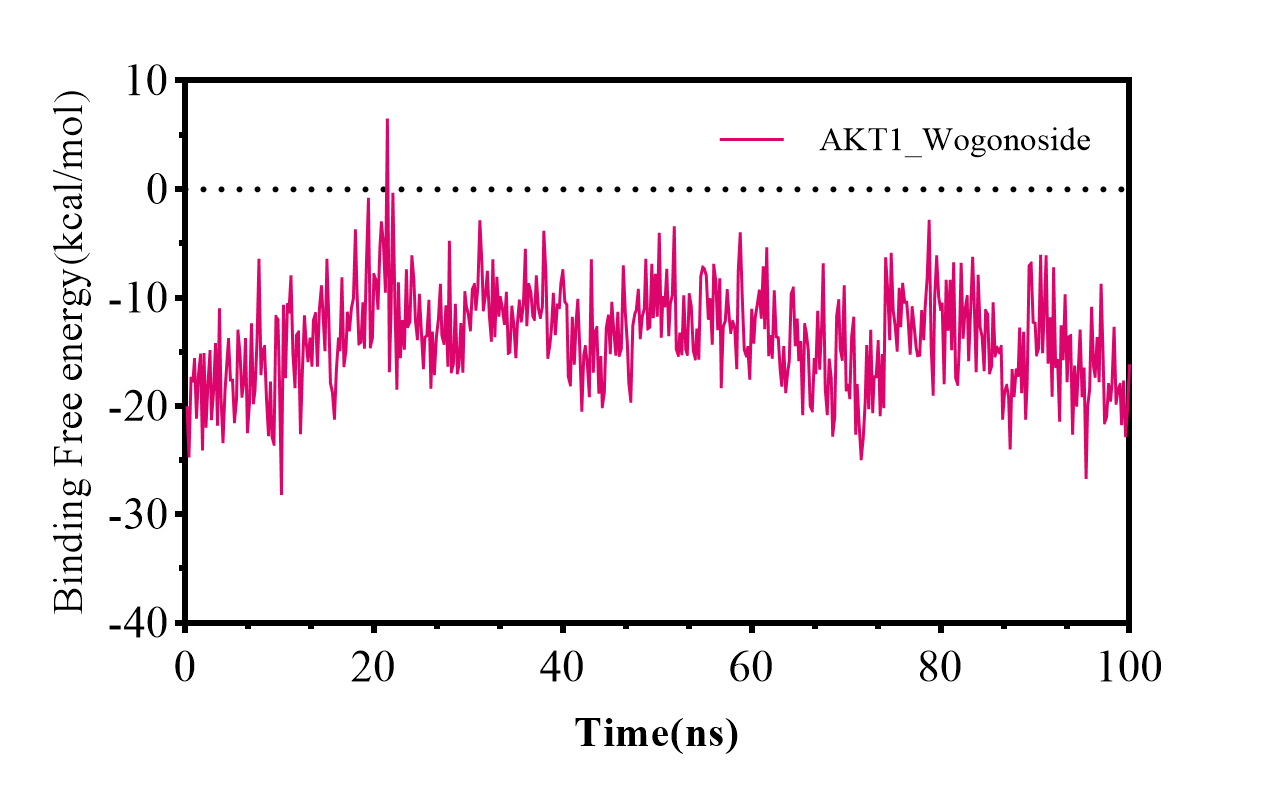

Supplement: Supplementary file 1 [file Data_Sheet_1.zip › MD Replicate2/MD Replicate2 BindingFreeEnergy.tif]

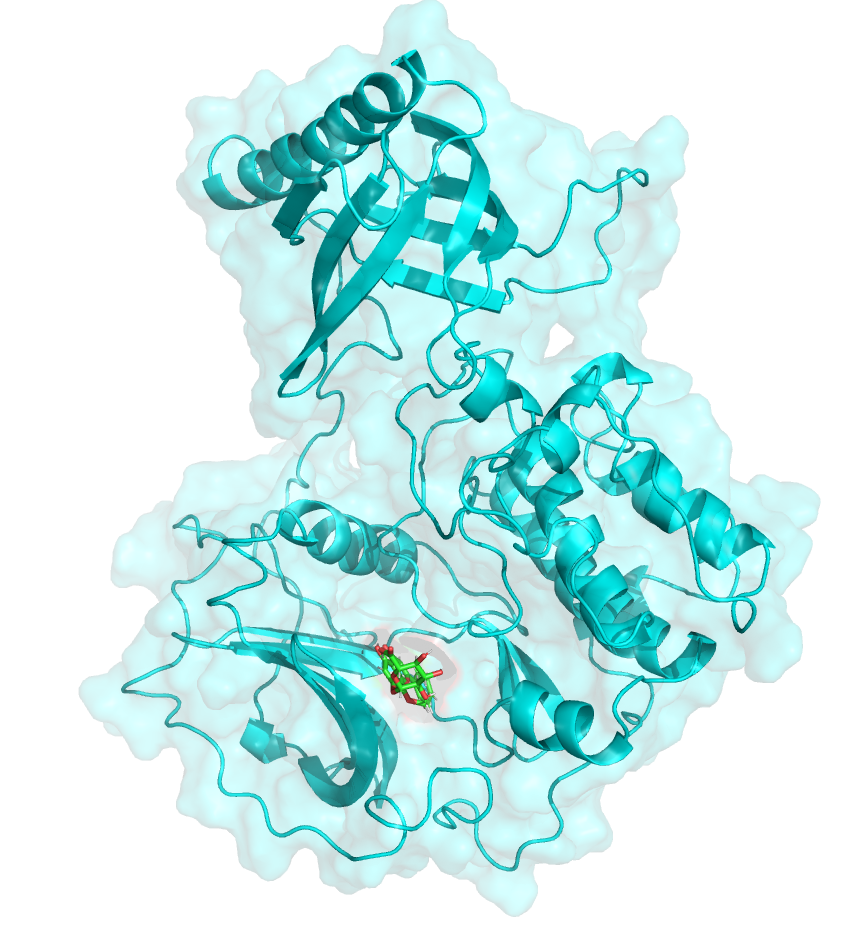

Supplement: Supplementary file 1 [file Data_Sheet_1.zip › MD Replicate2/MD Replicate2 ComplexStructure.tif]

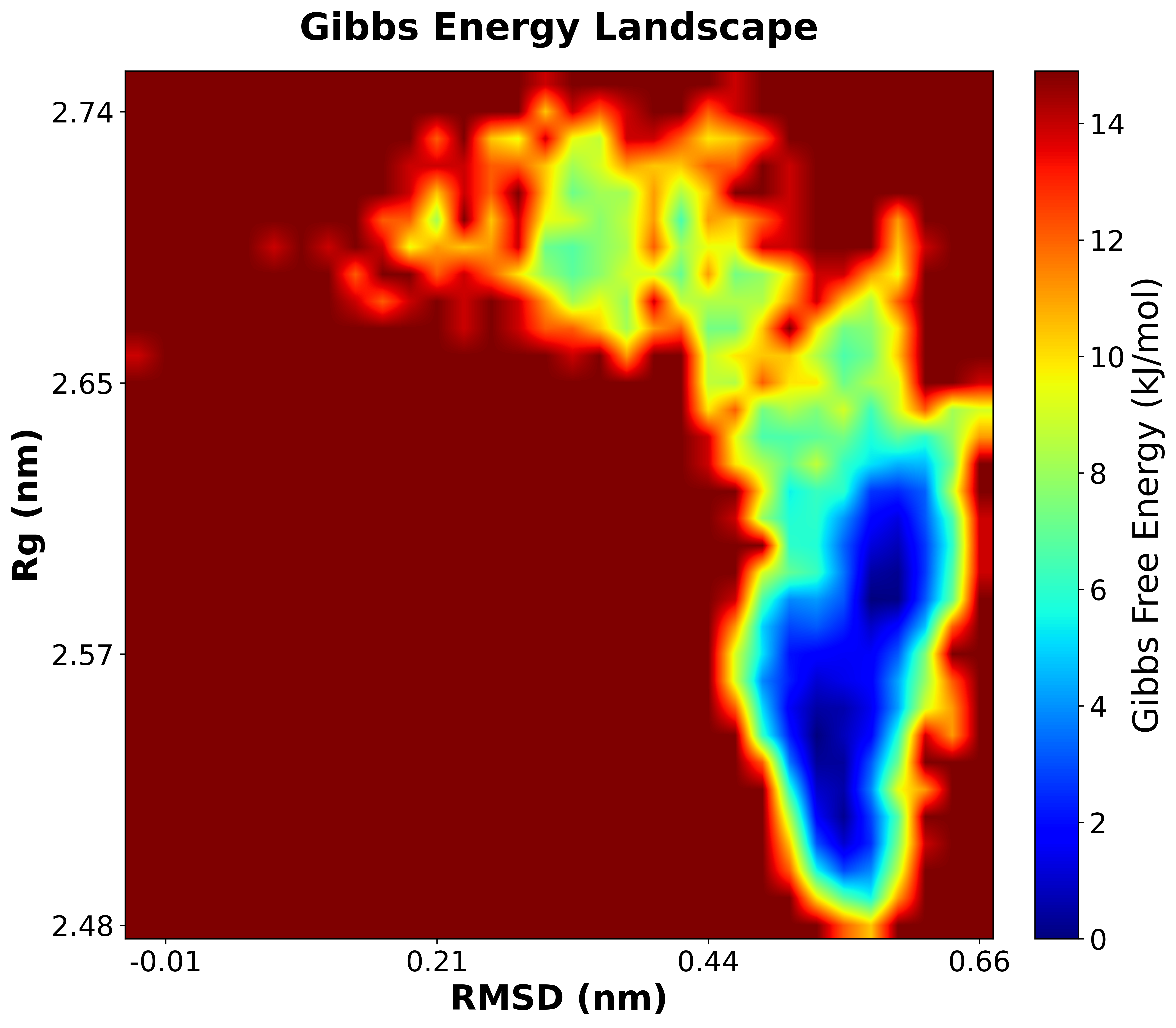

Supplement: Supplementary file 1 [file Data_Sheet_1.zip › MD Replicate2/MD Replicate2 FEL 2D.tif]

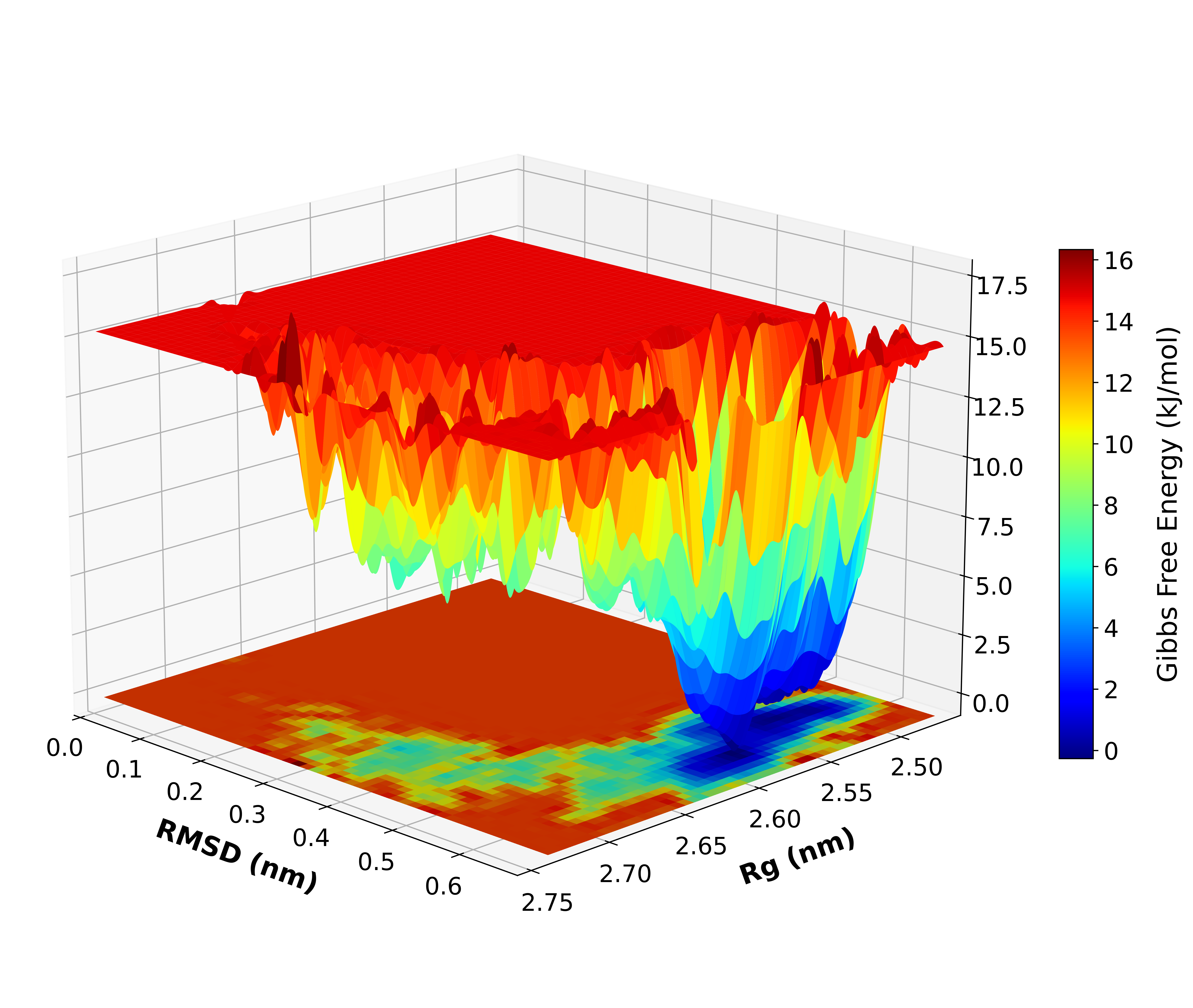

Supplement: Supplementary file 1 [file Data_Sheet_1.zip › MD Replicate2/MD Replicate2 FEL 3D.tif]

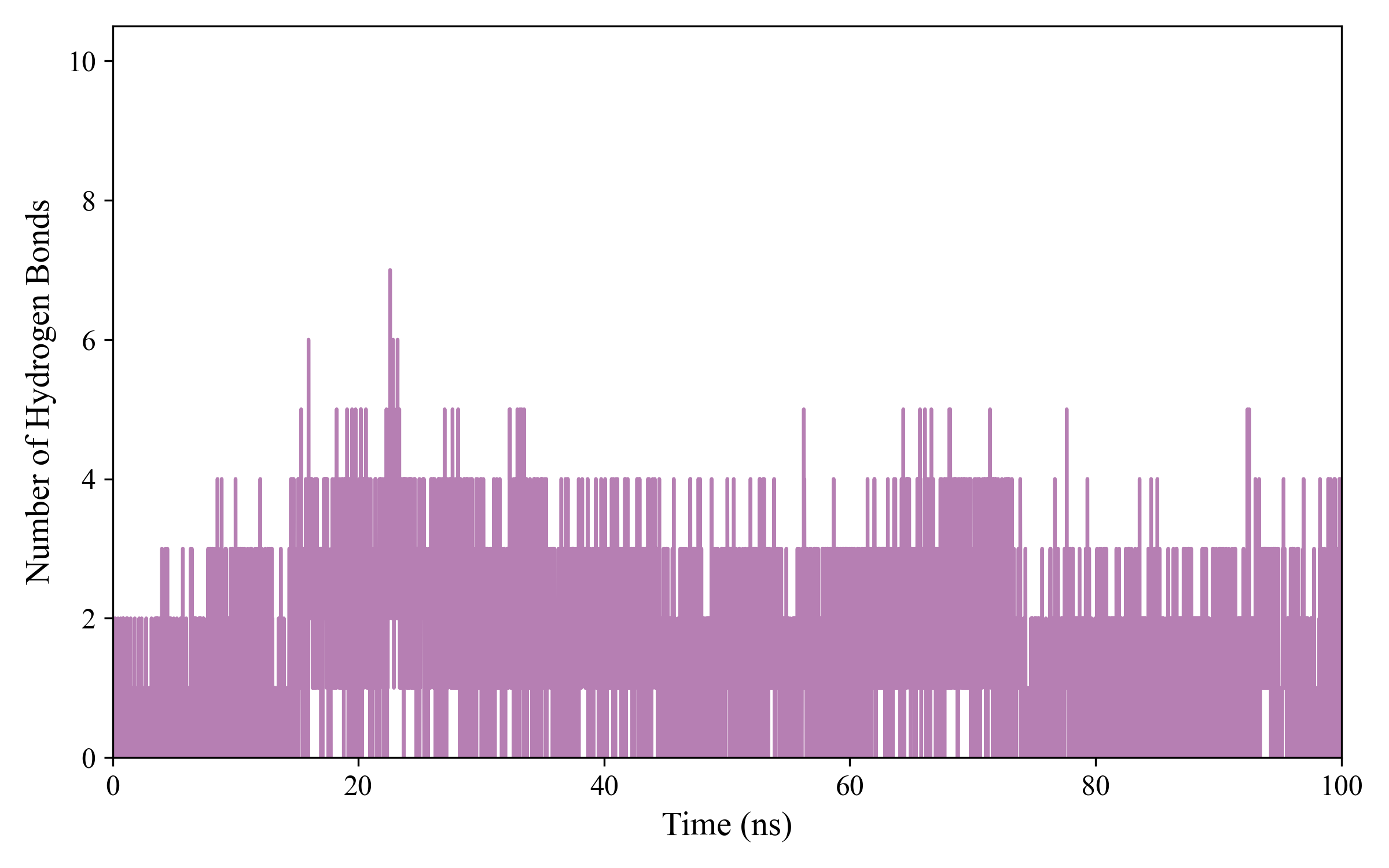

Supplement: Supplementary file 1 [file Data_Sheet_1.zip › MD Replicate2/MD Replicate2 HydrogenBonds.tif]

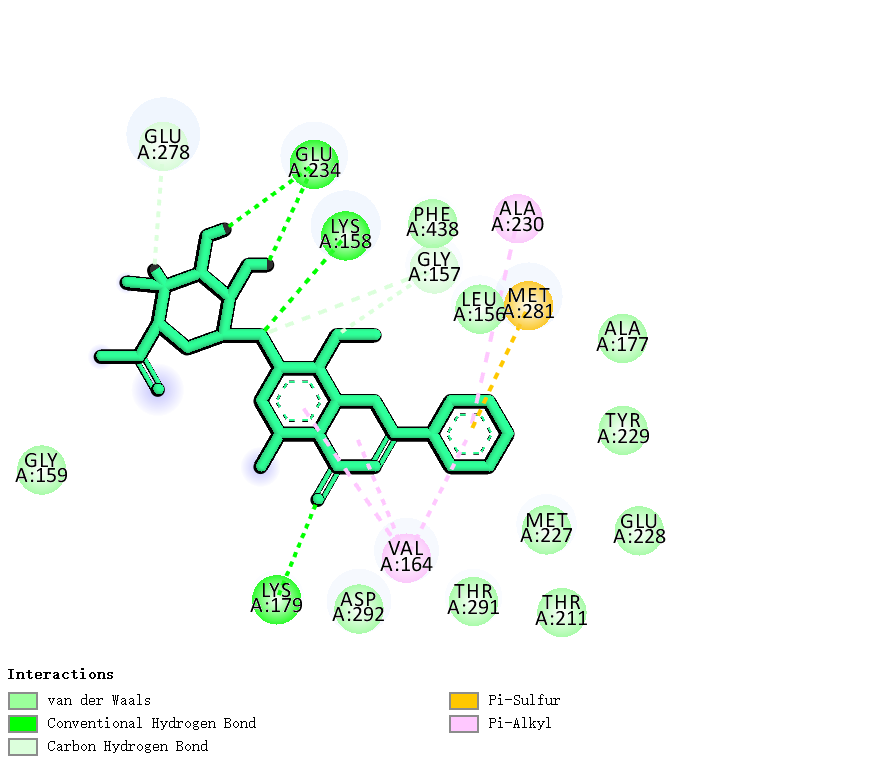

Supplement: Supplementary file 1 [file Data_Sheet_1.zip › MD Replicate2/MD Replicate2 MinimumEnergyStructure.tif]

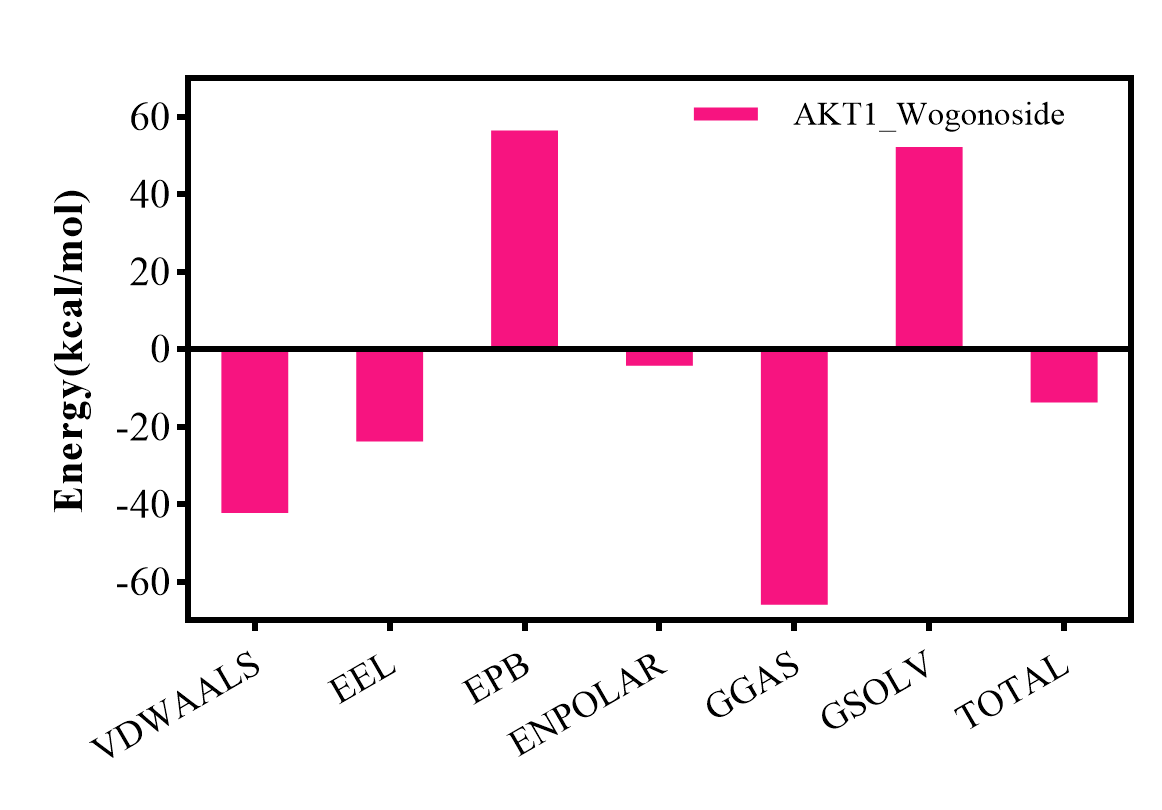

Supplement: Supplementary file 1 [file Data_Sheet_1.zip › MD Replicate2/MD Replicate2 MMPBSA EnergyComponents.tif]

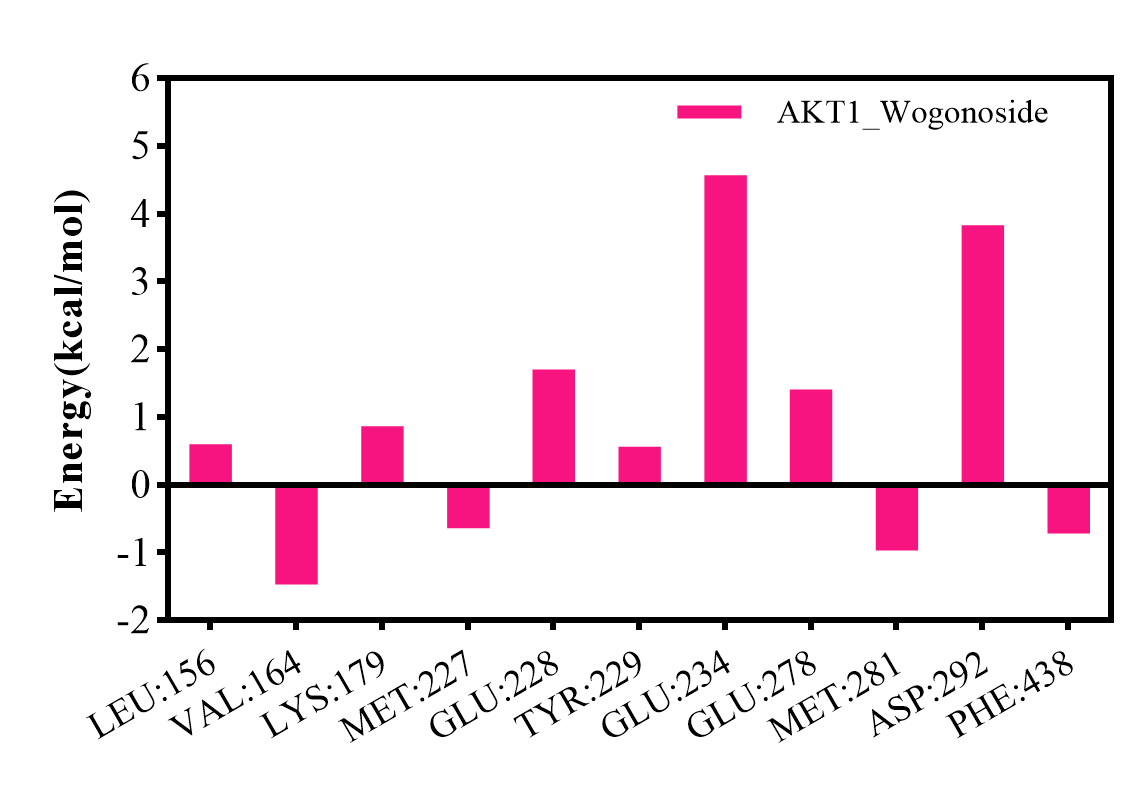

Supplement: Supplementary file 1 [file Data_Sheet_1.zip › MD Replicate2/MD Replicate2 PerResidueEnergyDecomposition.tif]

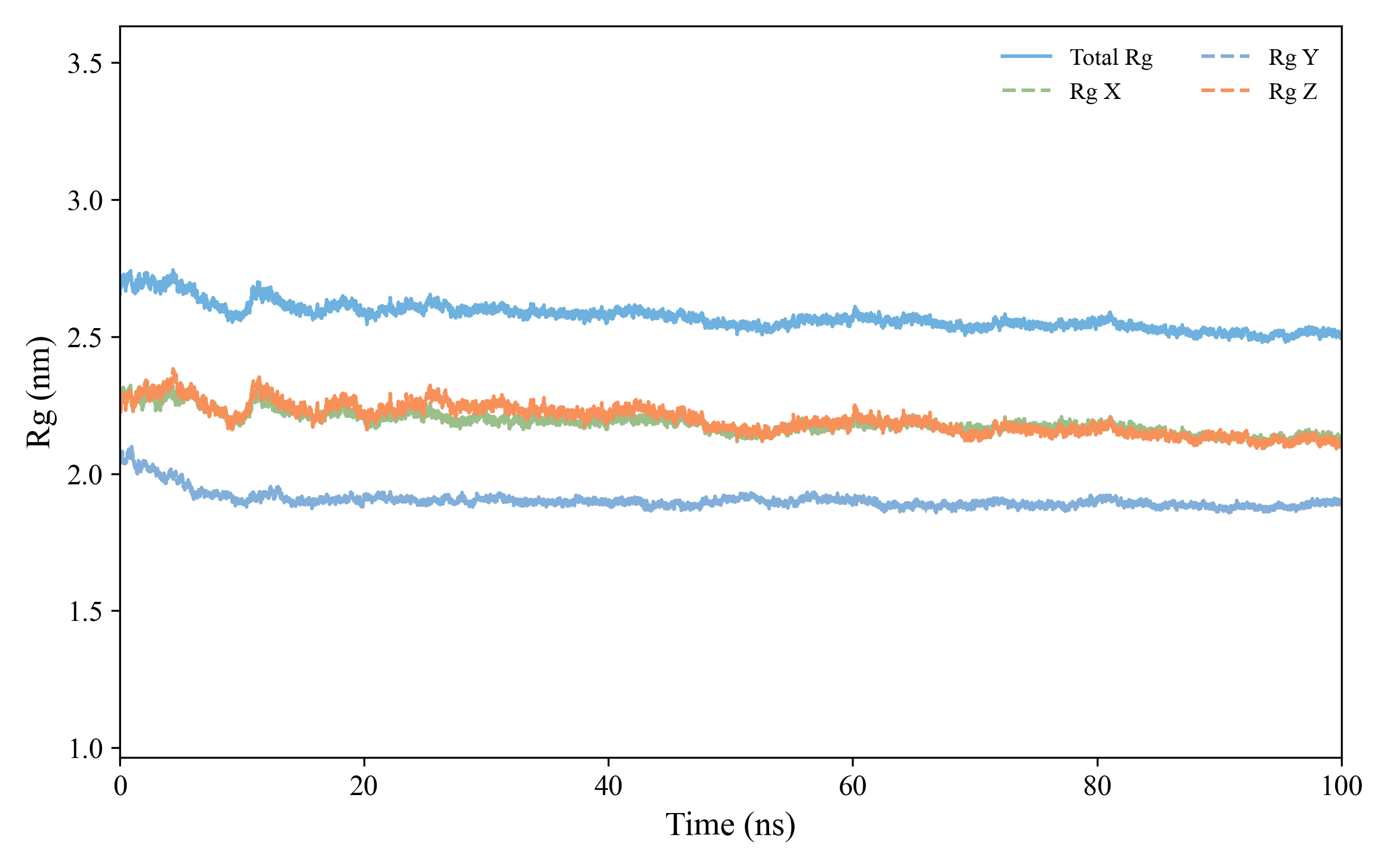

Supplement: Supplementary file 1 [file Data_Sheet_1.zip › MD Replicate2/MD Replicate2 Rg.tif]

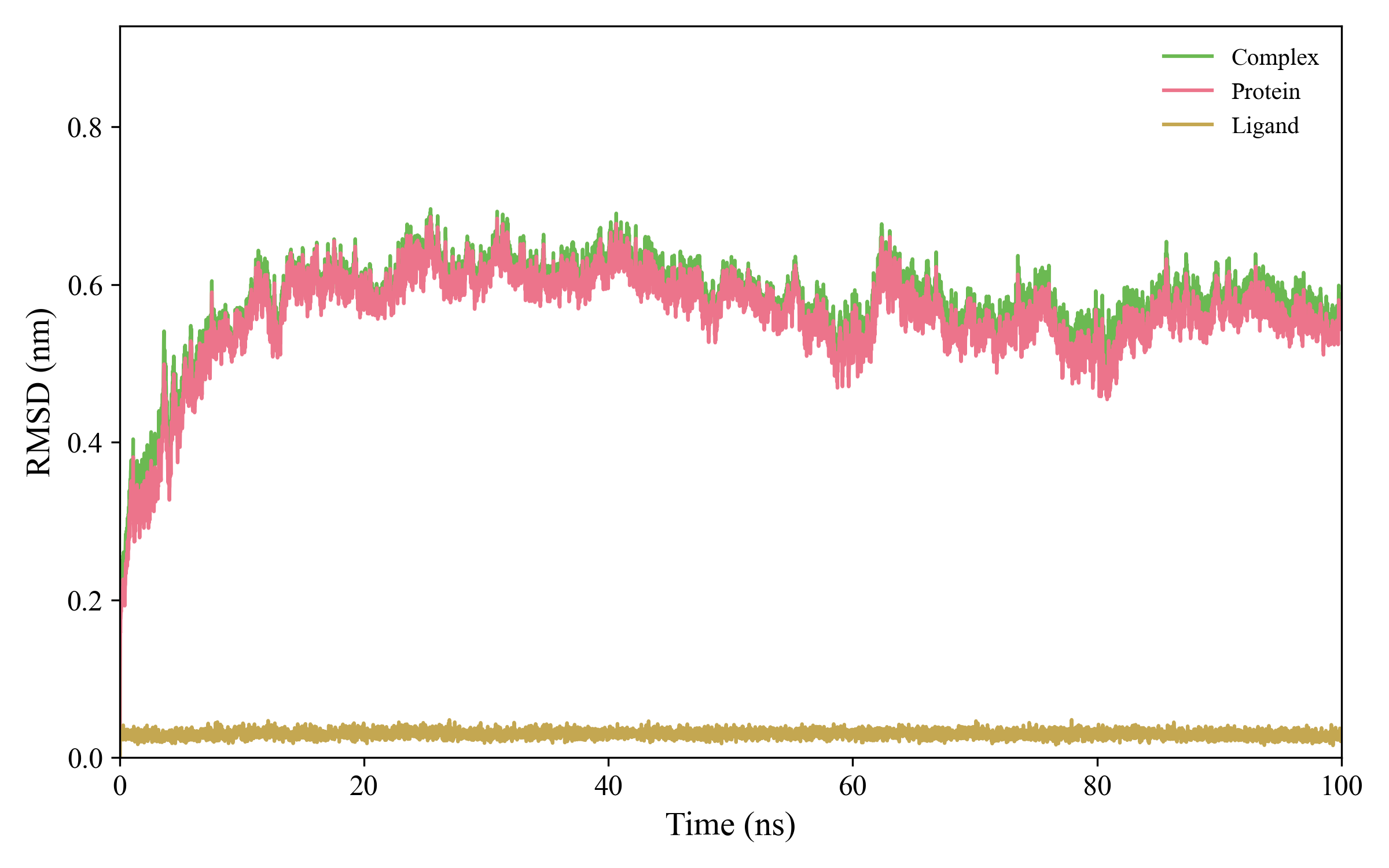

Supplement: Supplementary file 1 [file Data_Sheet_1.zip › MD Replicate2/MD Replicate2 RMSD.tif]

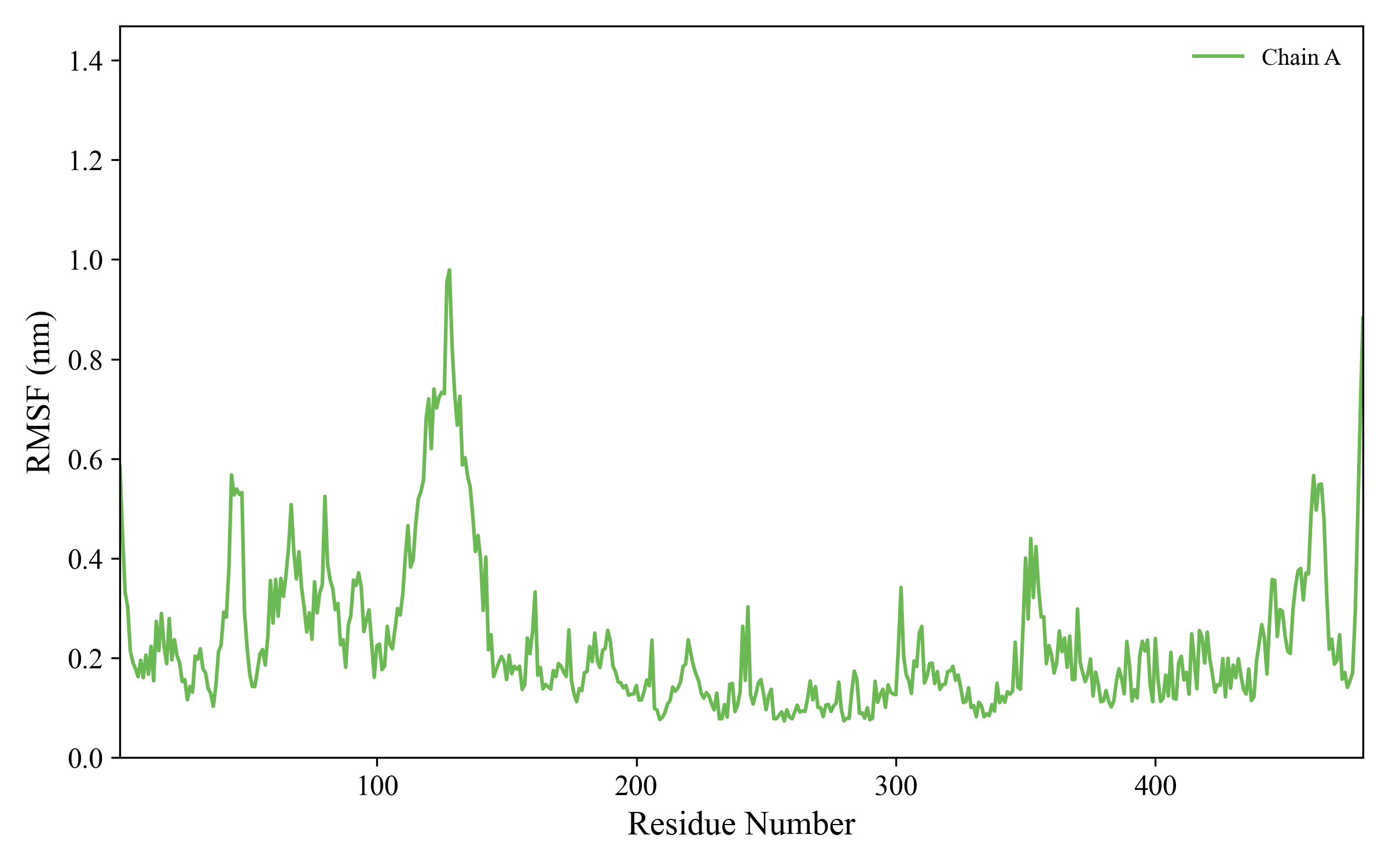

Supplement: Supplementary file 1 [file Data_Sheet_1.zip › MD Replicate2/MD Replicate2 RMSF.tif]

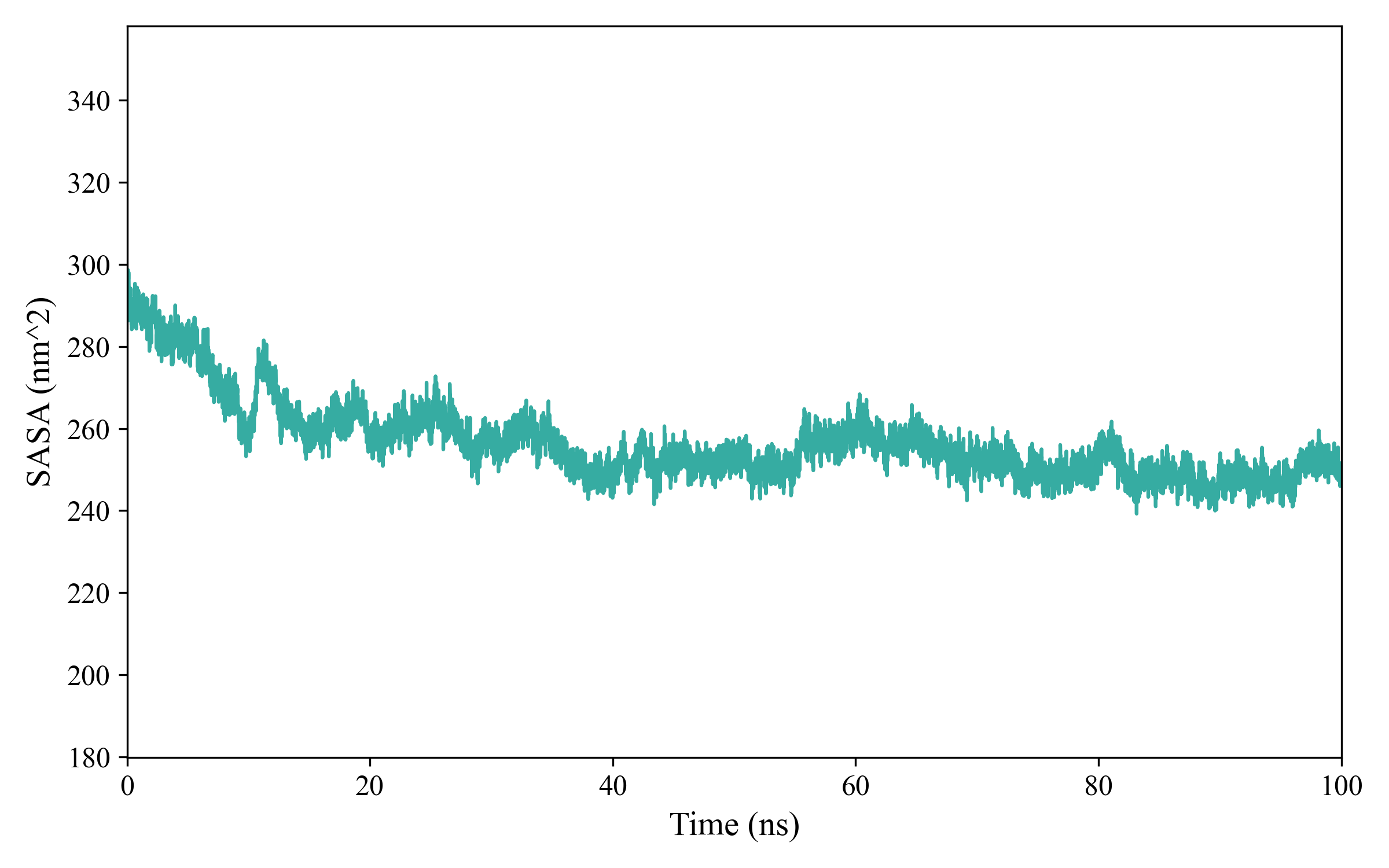

Supplement: Supplementary file 1 [file Data_Sheet_1.zip › MD Replicate2/MD Replicate2 SASA.tif]

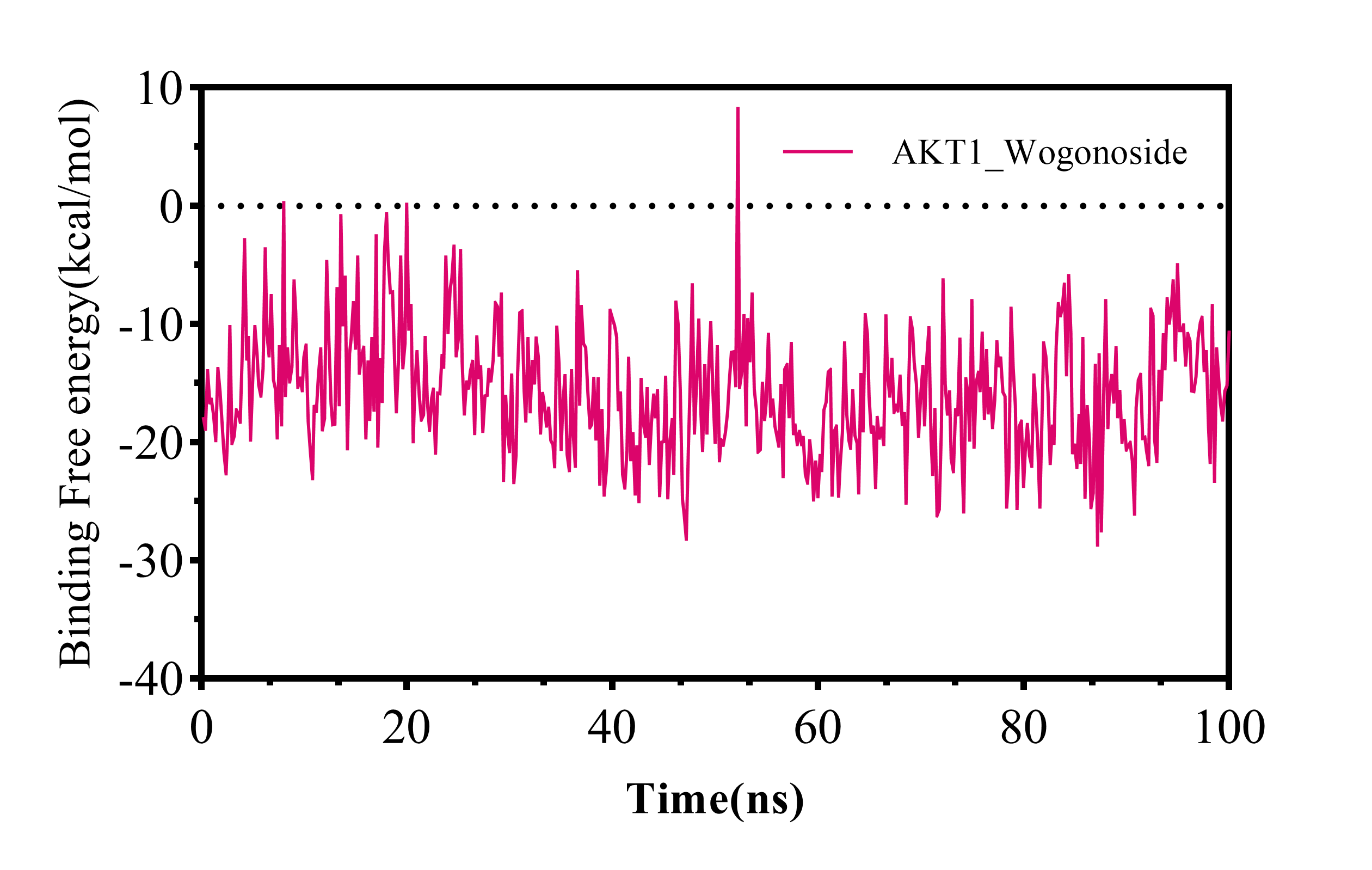

Supplement: Supplementary file 2 [file Data_Sheet_2.zip › MD Replicate3/MD Replicate3 BindingFreeEnergy.tif]

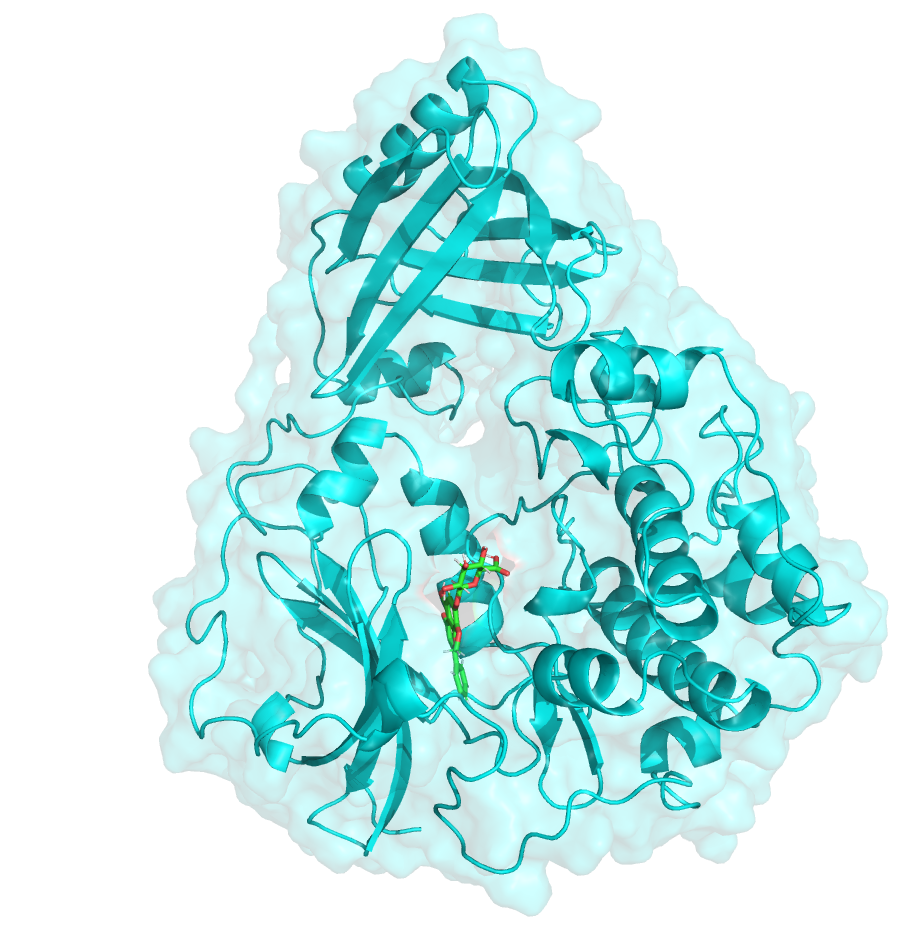

Supplement: Supplementary file 2 [file Data_Sheet_2.zip › MD Replicate3/MD Replicate3 ComplexStructure.tif]

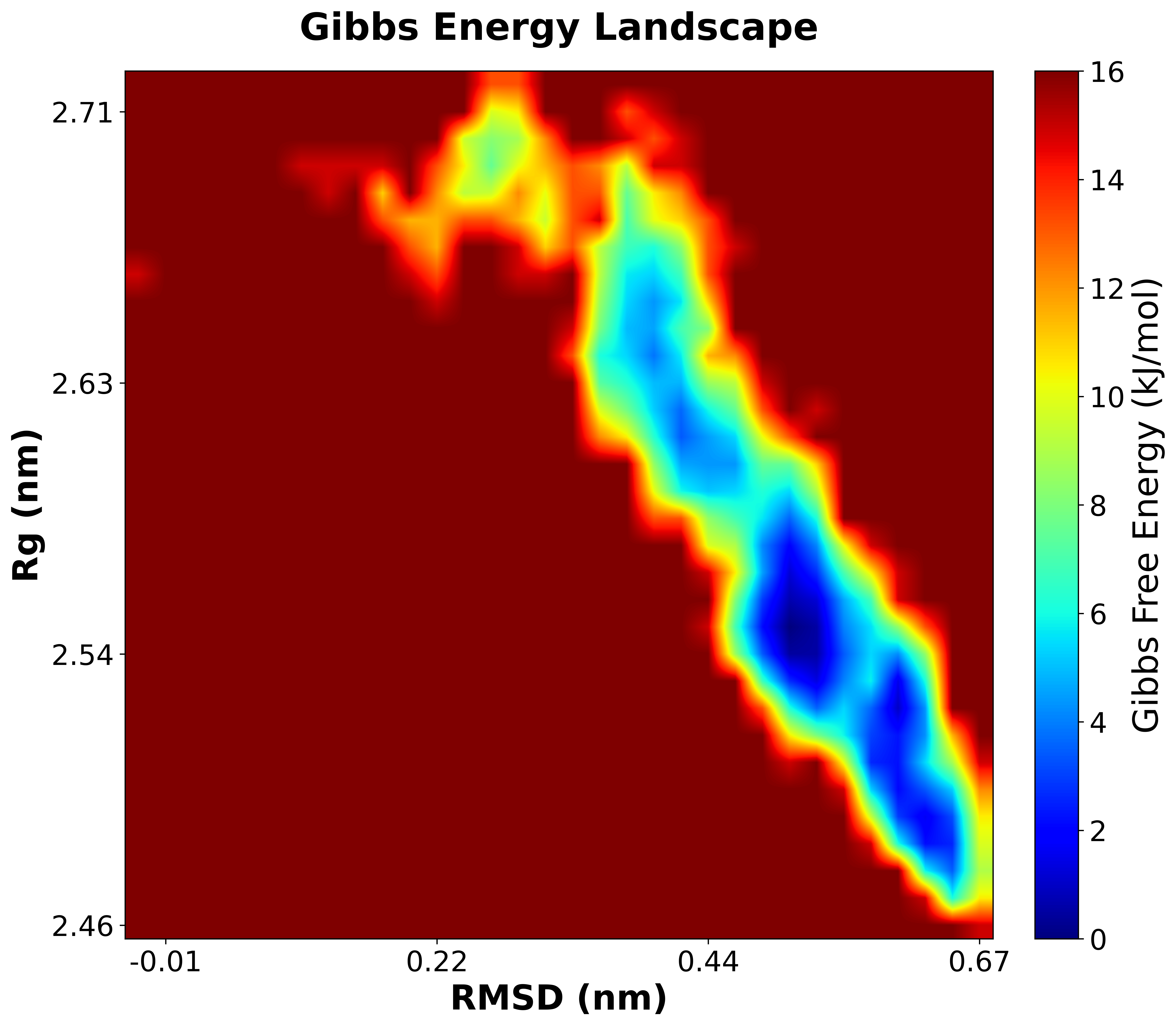

Supplement: Supplementary file 2 [file Data_Sheet_2.zip › MD Replicate3/MD Replicate3 FEL 2D.tif]

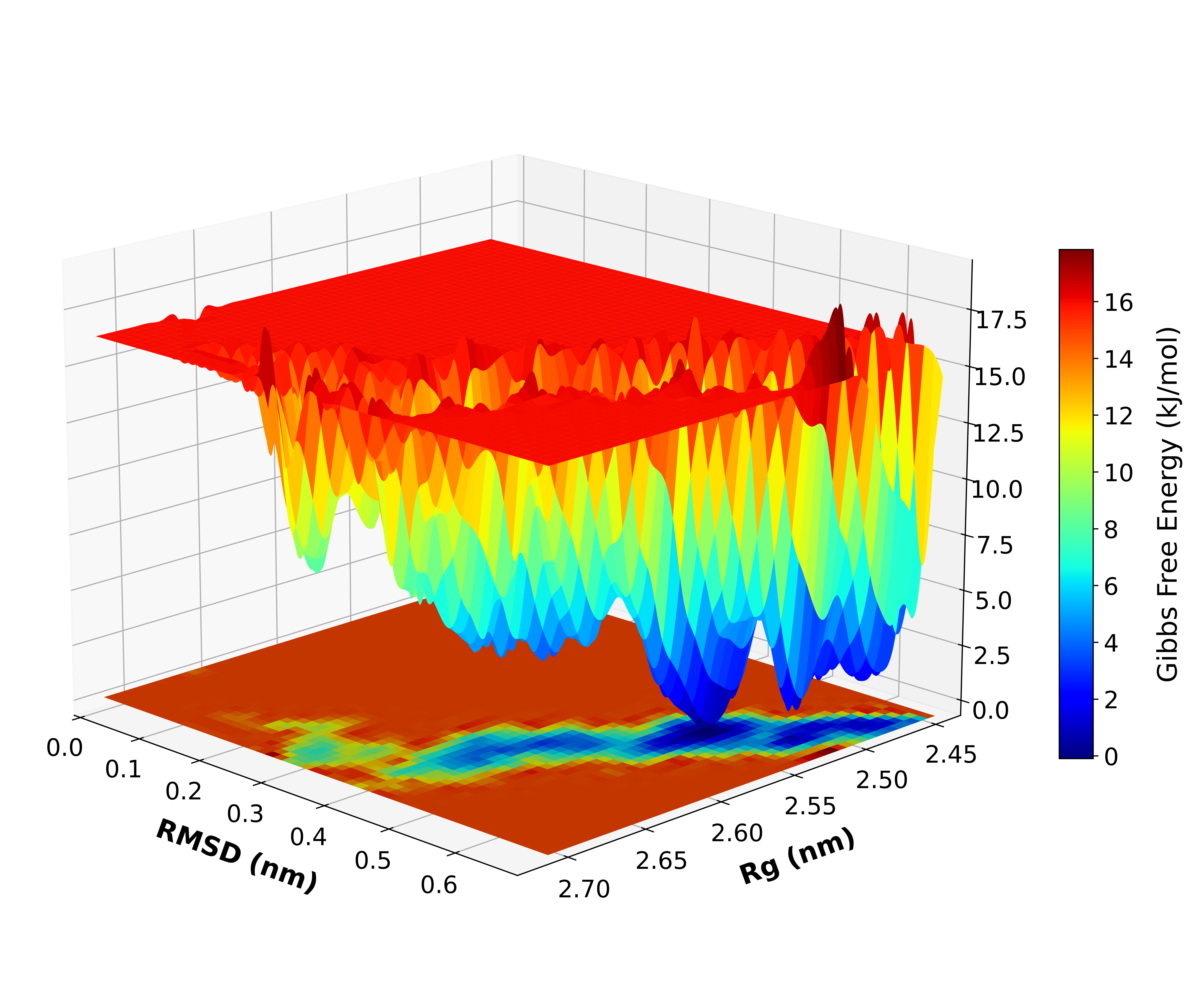

Supplement: Supplementary file 2 [file Data_Sheet_2.zip › MD Replicate3/MD Replicate3 FEL 3D.tif]

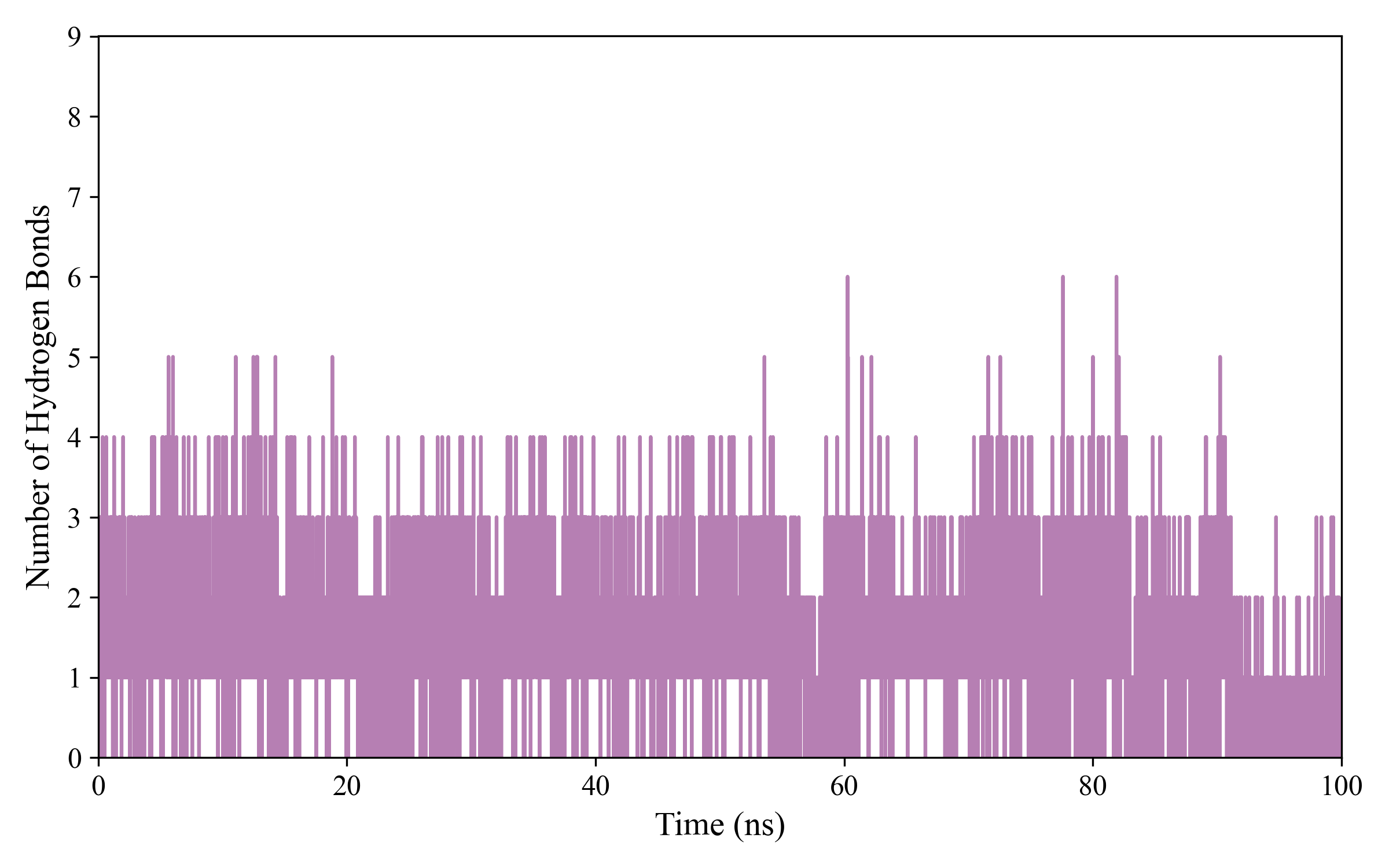

Supplement: Supplementary file 2 [file Data_Sheet_2.zip › MD Replicate3/MD Replicate3 HydrogenBonds.tif]

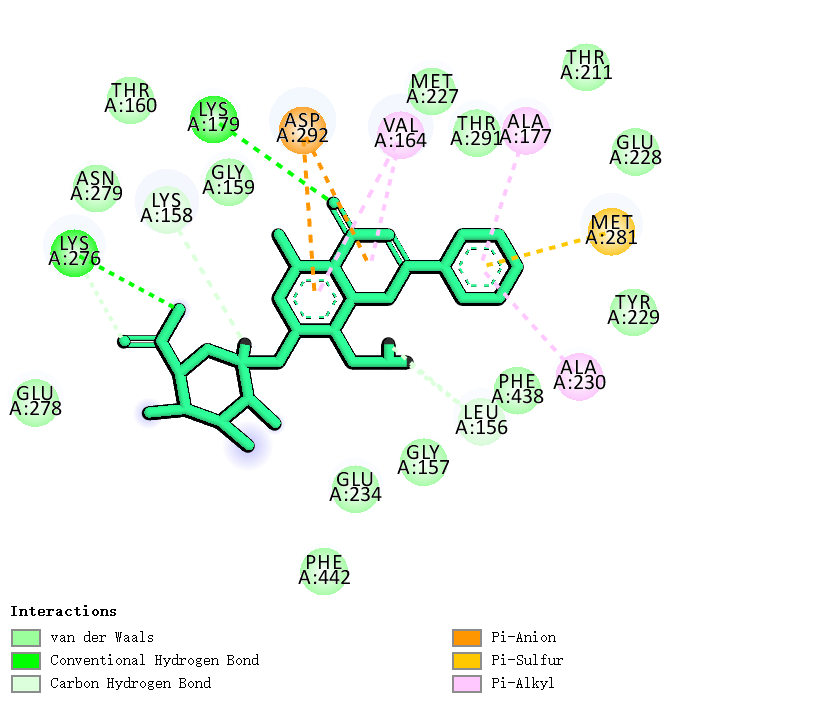

Supplement: Supplementary file 2 [file Data_Sheet_2.zip › MD Replicate3/MD Replicate3 MinimumEnergyStructure.tif]

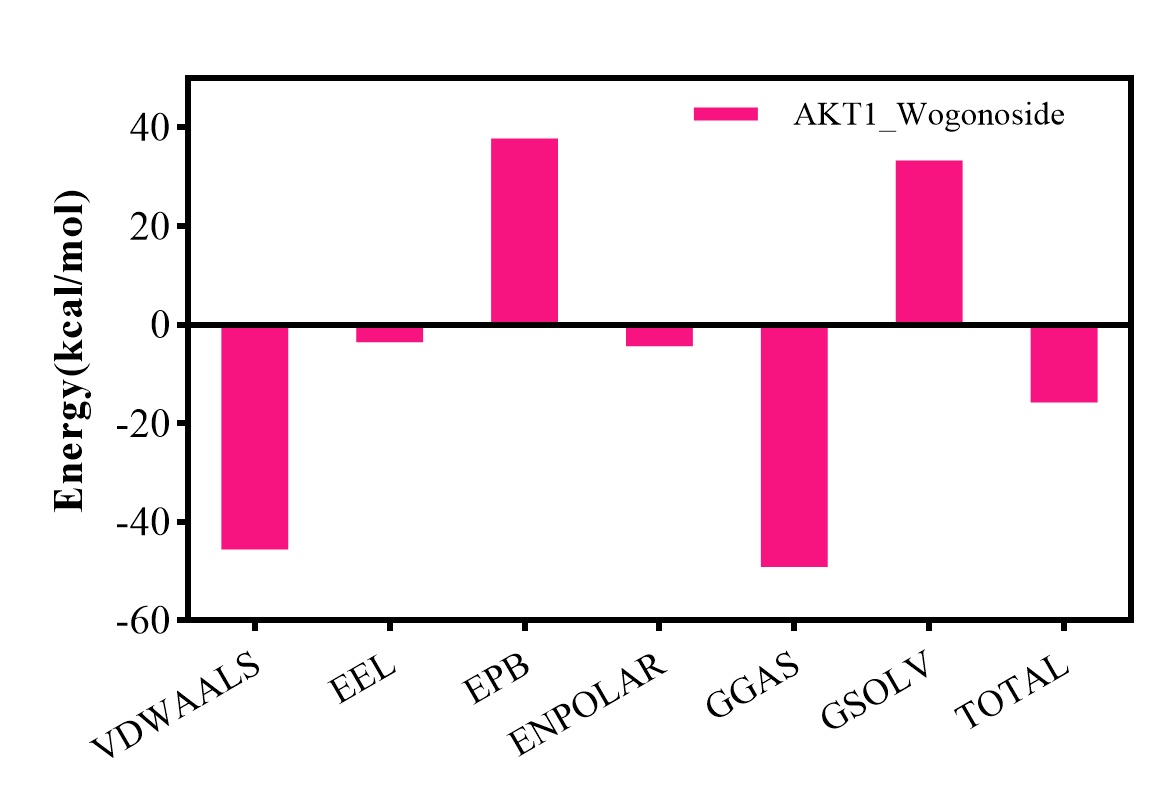

Supplement: Supplementary file 2 [file Data_Sheet_2.zip › MD Replicate3/MD Replicate3 MMPBSA EnergyComponents.tif]

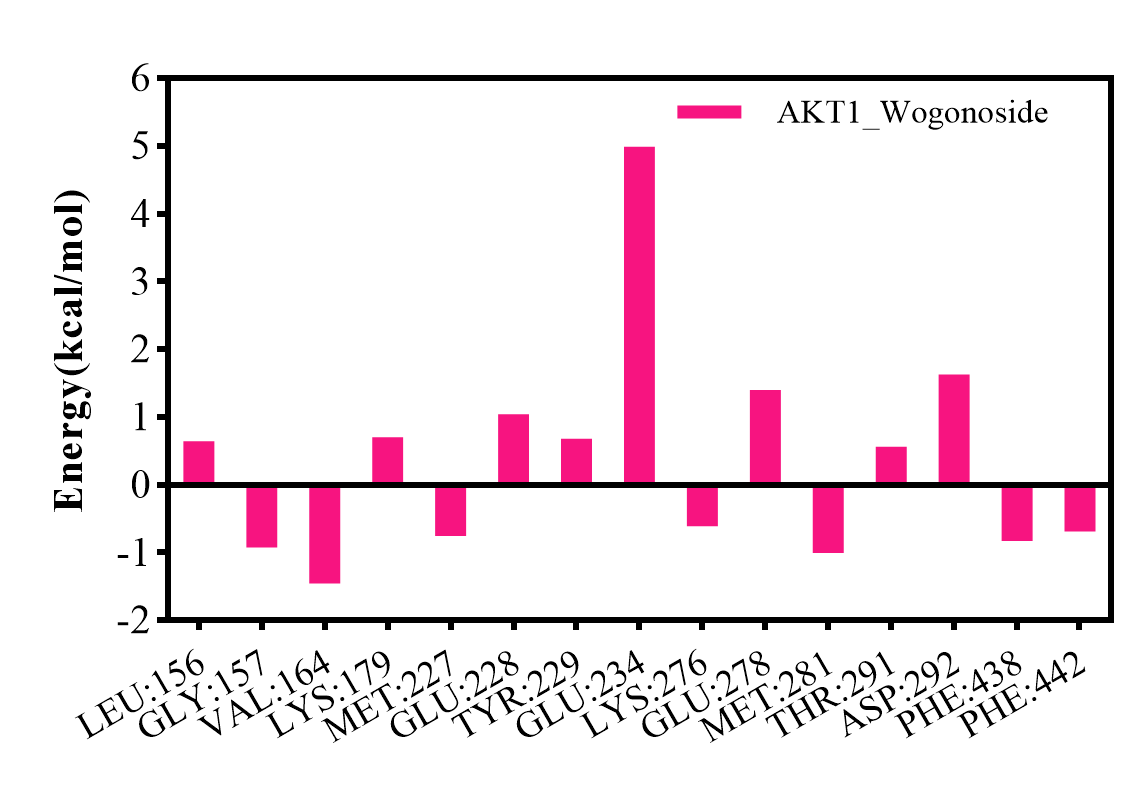

Supplement: Supplementary file 2 [file Data_Sheet_2.zip › MD Replicate3/MD Replicate3 PerResidueEnergyDecomposition.tif]

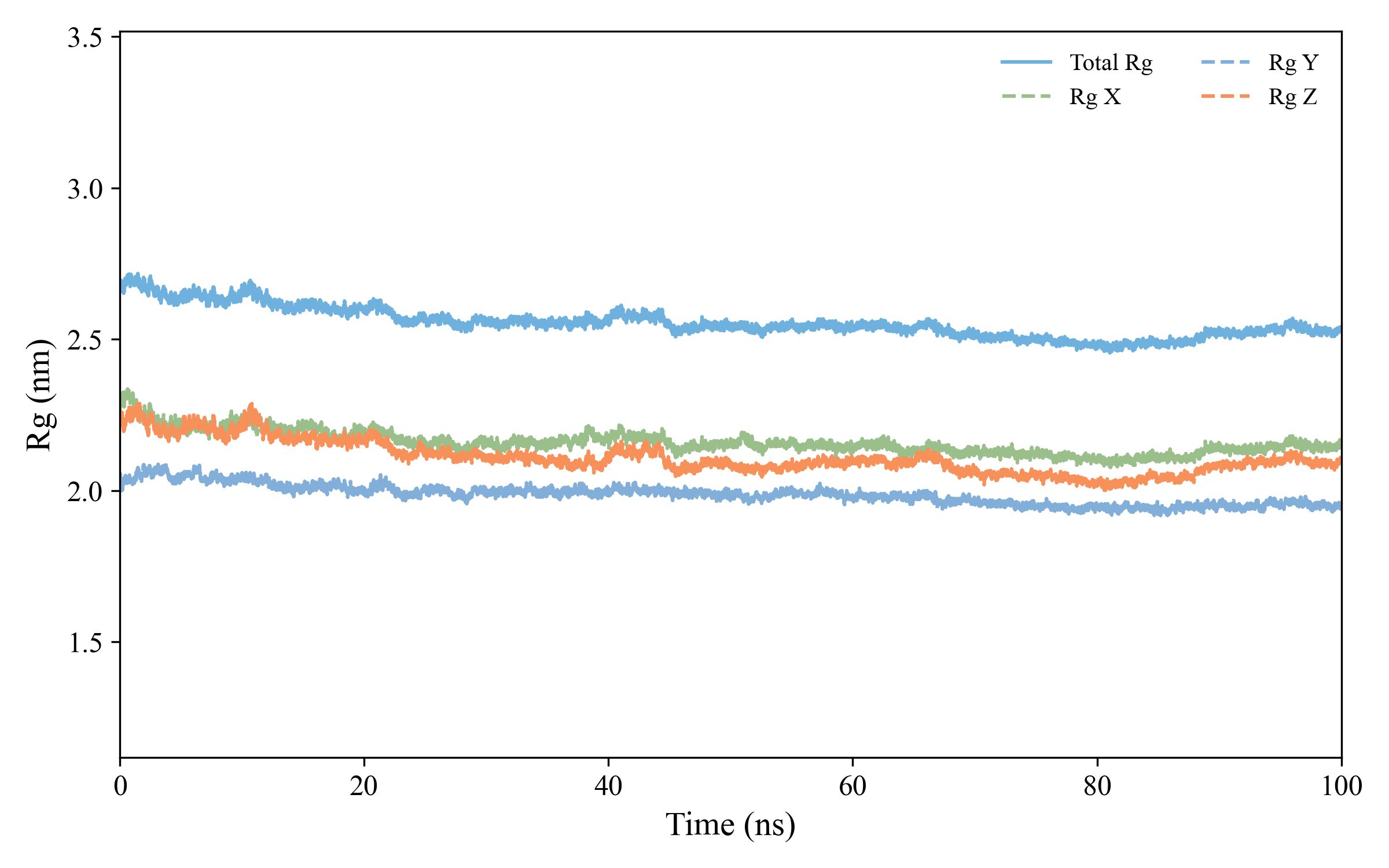

Supplement: Supplementary file 2 [file Data_Sheet_2.zip › MD Replicate3/MD Replicate3 Rg.tif]

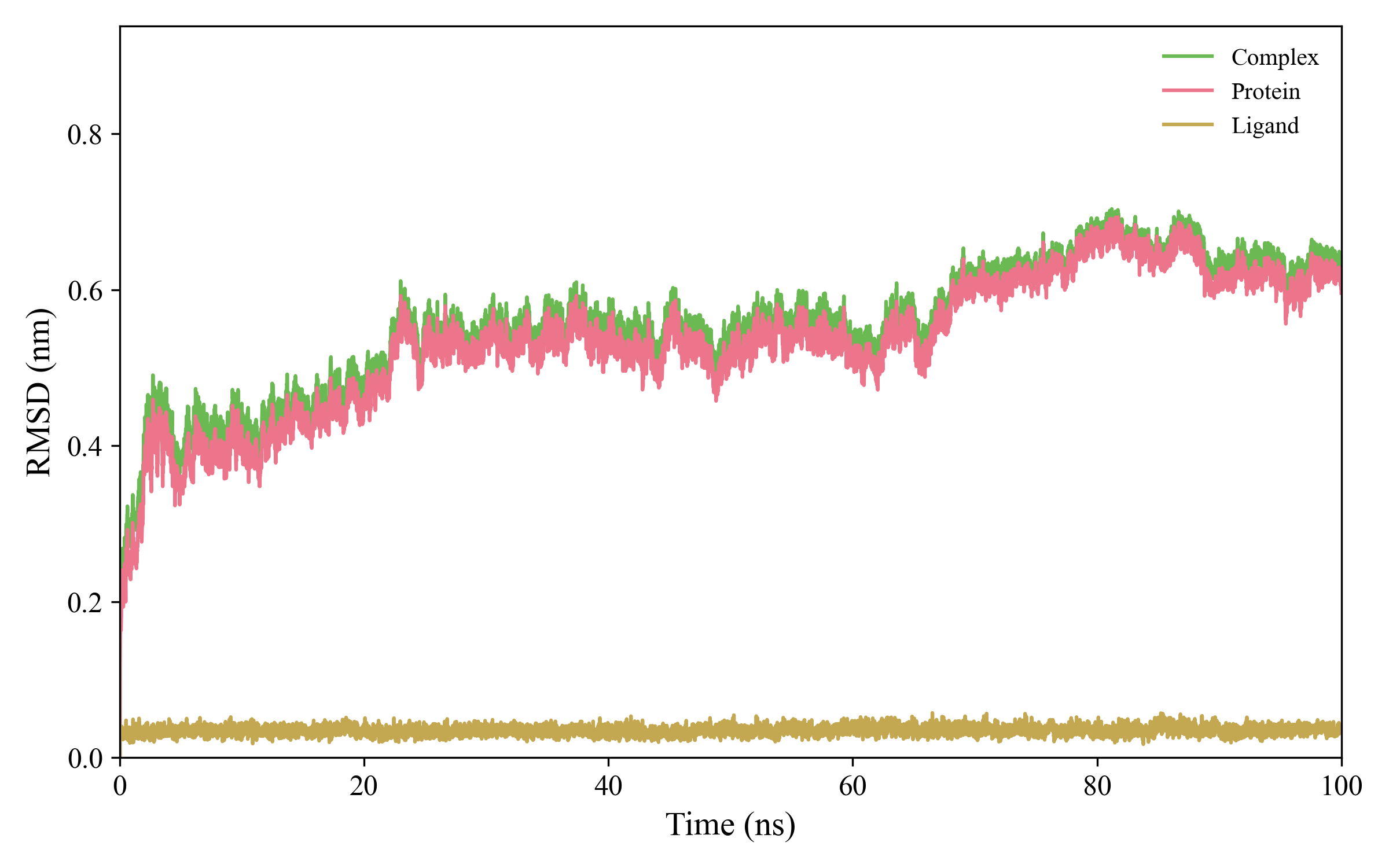

Supplement: Supplementary file 2 [file Data_Sheet_2.zip › MD Replicate3/MD Replicate3 RMSD.tif]

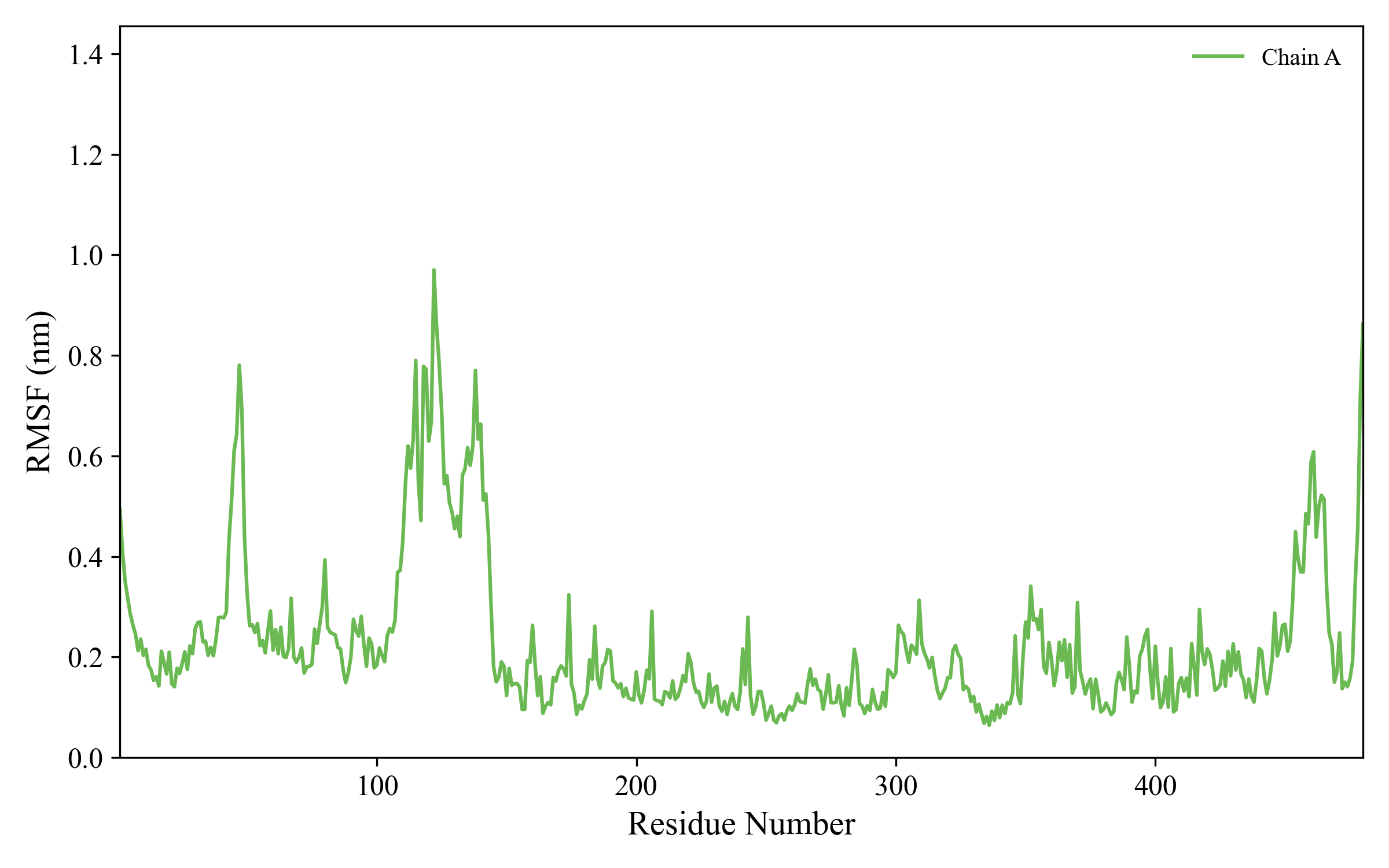

Supplement: Supplementary file 2 [file Data_Sheet_2.zip › MD Replicate3/MD Replicate3 RMSF.tif]

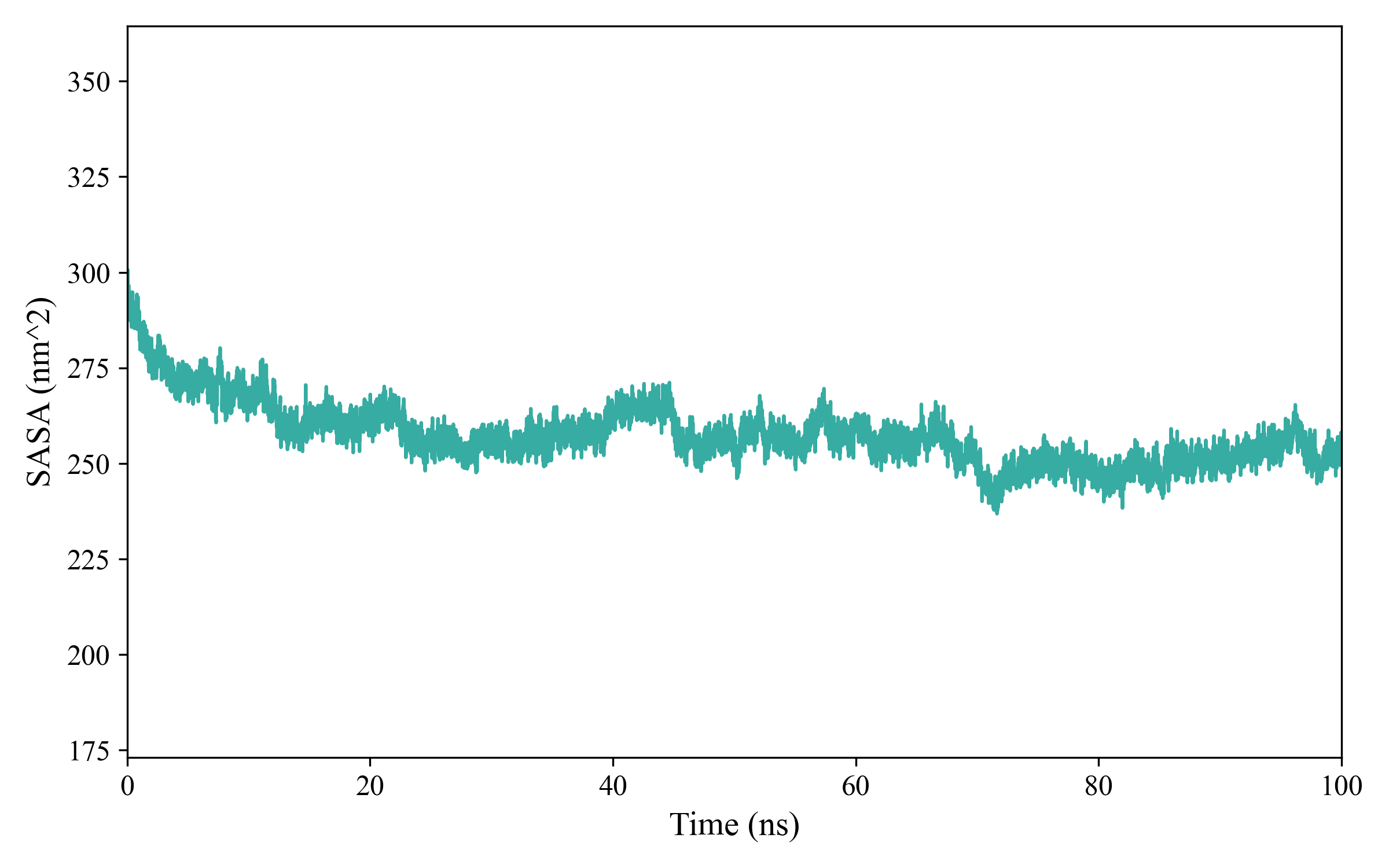

Supplement: Supplementary file 2 [file Data_Sheet_2.zip › MD Replicate3/MD Replicate3 SASA.tif]
